# The Zinc Transporter SLC39A13/ZIP13 Is Required for Connective Tissue Development; Its Involvement in BMP/TGF-β Signaling Pathways

**DOI:** 10.1371/journal.pone.0003642

**Published:** 2008-11-05

**Authors:** Toshiyuki Fukada, Natacha Civic, Tatsuya Furuichi, Shinji Shimoda, Kenji Mishima, Hiroyuki Higashiyama, Yayoi Idaira, Yoshinobu Asada, Hiroshi Kitamura, Satoru Yamasaki, Shintaro Hojyo, Manabu Nakayama, Osamu Ohara, Haruhiko Koseki, Heloisa G. dos Santos, Luisa Bonafe, Russia Ha-Vinh, Andreas Zankl, Sheila Unger, Marius E. Kraenzlin, Jacques S. Beckmann, Ichiro Saito, Carlo Rivolta, Shiro Ikegawa, Andrea Superti-Furga, Toshio Hirano

**Affiliations:** 1 Laboratory for Cytokine Signaling, RIKEN Research Center for Allergy and Immunology, Tsurumi, Yokohama, Kanagawa, Japan; 2 Department of Allergy and Immunology, Osaka University Graduate School of Medicine, Osaka University, Osaka, Japan; 3 Department of Medical Genetics, University of Lausanne, Lausanne, Switzerland; 4 Laboratory of Bone and Joint Diseases, Center for Genomic Medicine, RIKEN, Minato-ku, Tokyo, Japan; 5 Department of Anatomy-1, Tsurumi University School of Dental Medicine, Tsurumi-ku, Yokohama, Kanagawa, Japan; 6 Department of Pathology, Tsurumi University School of Dental Medicine, Tsurumi-ku, Yokohama, Kanagawa, Japan; 7 Department of Pediatric Dentistry, Tsurumi University School of Dental Medicine, Tsurumi-ku, Yokohama, Kanagawa, Japan; 8 Laboratory for Immunogenomics, RIKEN Research Center for Allergy and Immunology, Tsurumi, Yokohama, Kanagawa, Japan; 9 Kazusa DNA Research Institute, Laboratory of Genome Technology, Kisarazu, Chiba, Japan; 10 Laboratory for Developmental Genetics, RIKEN Research Center for Allergy and Immunology, Tsurumi, Yokohama, Kanagawa, Japan; 11 Serviço de Genética Médica, Hospital S. Maria, Lisboa, Portugal; 12 Division of Molecular Pediatrics, Centre Hospitalier Universitaire Vaudois, Lausanne, Switzerland; 13 Department of Paediatrics and Adolescent Medicine, University of Freiburg, Freiburg, Germany; 14 Division of Endocrinology, Diabetes and Clinical Nutrition, University Hospital, Basel, Switzerland; 15 Service of Medical Genetics, Centre Hospitalier Universitaire Vaudois, Lausanne, Switzerland; 16 Laboratory of Developmental Immunology and the CREST Program of the Japan Science and Technology Agency, Graduate School of Frontier Biosciences, Graduate School of Medicine, and WPI Immunology Frontier Research Center, Osaka University, Suita, Osaka, Japan; Center for Genomic Regulation, Spain

## Abstract

**Background:**

Zinc (Zn) is an essential trace element and it is abundant in connective tissues, however biological roles of Zn and its transporters in those tissues and cells remain unknown.

**Methodology/Principal Findings:**

Here we report that mice deficient in Zn transporter Slc39a13/Zip13 show changes in bone, teeth and connective tissue reminiscent of the clinical spectrum of human Ehlers-Danlos syndrome (EDS). The *Slc39a13* knockout (*Slc39a13*-KO) mice show defects in the maturation of osteoblasts, chondrocytes, odontoblasts, and fibroblasts. In the corresponding tissues and cells, impairment in bone morphogenic protein (BMP) and TGF-β signaling were observed. Homozygosity for a SLC39A13 loss of function mutation was detected in sibs affected by a unique variant of EDS that recapitulates the phenotype observed in *Slc39a13*-KO mice.

**Conclusions/Significance:**

Hence, our results reveal a crucial role of SLC39A13/ZIP13 in connective tissue development at least in part due to its involvement in the BMP/TGF-β signaling pathways. The *Slc39a13*-KO mouse represents a novel animal model linking zinc metabolism, BMP/TGF-β signaling and connective tissue dysfunction.

## Introduction

Zn is an essential trace element [1] and its homeostasis in the single cell and in whole organisms is tightly controlled by two major families of Zn transporters, Zn importers (SLC39s/ZIPs) [2] and exporters (SLC30s/ZnTs) [3], and Zn-binding proteins metallothioneins (MTs) [4]. Mice strains carrying mutations in genes related to zinc metabolism show various defects of development [5], [6]. Children affected by the recessive condition acrodermatitis enteropathica (AE) have low serum concentrations of Zn because of mutations in the intestinal Zn transporter SLC39A4/ZIP4. These patients suffer from severe skin disease and frequent infections [7], [8]. Zn is essential for the function of molecules with domains such as Zn-finger, Ring-finger and LIM domains [9], [10]. In addition, Zn has been implicated as signaling molecule or as affecting intracellular signaling pathways [5]. A nematode ZnT1 orthologue, CDF1, positively affects Ras-ERK signal transduction [11]. Slc39a7/Zip7 was found to affect EGF/IGF signaling and tamoxifen resistancy of breast cancer cells [12]. Slc39a6/Zip6/Liv1 controls the nuclear localization of the Zn-finger transcription factor Snail [13]. Extracellular signals, such as toll-like receptor 4 (TLR4)- and FcεR1-mediated stimulation, induce the change of intracellular level of free Zn in dendritic cells and mast cells, respectively, and this in turn controls the biological activities of extracellular stimuli [14], [15]. These reports all support the idea that Zn is an intracellular signaling molecule and lead to the prediction that Zn transporters have roles not only for maintaining Zn homeostasis, but also for mediating intracellular signaling events [5].

In Zn-deficient conditions, bone growth retardation and increase of skin fragility are commonly observed [16], [17]. Indeed, Zn concentrations are high in bone, cartilage, and teeth[18], and Zn may play a role in bone metabolism by stimulating bone formation and mineralization [19]. Zn is also condensed in epidermal and dermal cells and in their extracellular matrix (ECM) [20], [21]. The *MT*-null mice show low concentration of Zn in skin, and the epidermis fails to exhibit hyperplasia [22]. These evidence suggests an important role of Zn in development of both hard and soft connective tissues, which require well-coordinated local paracrine regulators such as BMP and TGF-β to be developed [23], [24], [25], [26]. Human genetics studies revealed that they play a pivotal role for connective tissue development [27], [28].

The Ehlers-Danlos syndrome (EDS) is a group of genetic disorders affecting connective tissues. Several types are distinguished based on clinical features, inheritance pattern, and molecular basis [29]. Many of them originate from changes in the primary structure or posttranslational modifications of fibrillar collagens [30]. While our studies were in progress, a mutation in *SLC39A13* was found in two families with a newly recognized variant of EDS [31], similar to the one we observed in two sibs (see below). In that work, emphasis is given to the impairment in collagen lysyl hydroxylation, a feature observed also in our patients, but no explanation is given for the short stature and other phenotypic features observed, which clearly distinguish the novel EDS type from EDS VIA (procollagen lysyl hydroxylase deficiency). Other EDS types, such as EDS type VIB or EDS type VIID, and related conditions such as the Brittle Cornea Syndrome [32] are still awaiting molecular elucidation; intriguingly, the causative gene for the Brittle Cornea Syndrome has been found to be a Zn-finger gene [33].

Here we report that knockout of *Slc39a13* in mice results in a generalized skeletal and connective tissue disorder, and that a homozygous loss of function mutation in *SLC39A13* is found in a unique type of the EDS in human subjects. In addition, Slc39a13 controls intracellular Zn distribution and is involved in BMP and TGF-β signal transduction pathways in connective tissues. Thus, our results allow to establish a genetic and functional link between the Zn transporter Slc39a13 and connective tissue development, showing the usefulness of the *Slc39a13*-KO mouse as a novel animal model for human connective tissue diseases.

## Results

### Reduced osteogenesis and abnormal cartilage development in *Slc39a13*-KO mice

**Figure 1:**
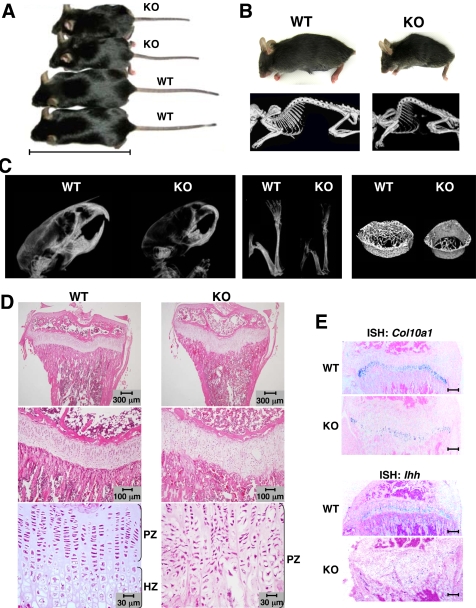
Growth retardation, kyphosis, osteopenia, and abnormal cartilage development in *Slc39a13*-KO mice. A. 5-week-old female wild-type and *Slc39a13*-KO mice. A bar indicates 10 cm. B. Kyphosis in *Slc39a13*-KO mouse. Appearance and radiographs of 5-week-old mice. C. Osteopenia of *Slc39a13*-KO mice. X-rays of skull (left), femur and tibia (middle) of 4-week-old mice. 3D-mCT of the tibial diaphysis (right). D. *Slc39a13*-KO mice show elongated growth plate with uncoordinated columnar formation, and decrease hypertrophic zone. Tibia from 4-week-old *Slc39a13*-KO mice and wild-type littermates stained with H&E. Large views of growth plate are shown in middle and lower panels. PZ, proliferative zone; HZ, hypertrophic zone. E. Gene expression of *Col10a1* and *Ihh* gene are diminished in growth plate of 4-week-old *Slc39a13*-KO mice. Bar indicates 300 µm. ISH images.

To examine the physiologic role of Slc39a13 *in vivo*, we performed gene depletion (Figures S1A and S1B). *Slc39a13*-KO mice showed growth retardation (Figures 1A and S2) and developed progressive kyphosis after 3 or 4 weeks of age (Figure 1B). *Slc39a13*-KO mice showed several abnormalities in bone: 1) Skull and long bones were radiolucent (Figure 1C, left and middle); 2) Decrease in cortical bone thickness, number of trabeculae, and bone volume (three-dimensional micro-computed tomography (3D-mCT), Figure 1C, right); 3) Decrease in bone mineral density (BMD) in skull, mandible, and cortex and cancellous of femur (Figure S3A); 4) Reduction of bone volume/total tissue volume (BV/TV) and osteoid thickness (O.Th) in tibial metaphyses (Figure S3B); 5) Double-labeling analysis using calcein, a marker of newly formed bone, revealed that calcification activities such as mineral apposition ratio (MAR) and bone formation rate (BFR/BS), an indicator of osteoblast function, were decreased (Figure S3C). However, eroded surface (ES/BS), the numbers of osteoclasts (N.Oc/B Pm), and osteoclast-covered bone surface (Oc.S/BS) were essentially equivalent to those in wild-type littermates (Figure S3D). These data indicated that *Slc39a13*-KO mice had reduced osteoblast activity with little alteration of osteoclast activity.

In addition to decrease of bone mass, *Slc39a13*-KO mice had a significant reduction in long bone length (Figure S4A). The width of growth plate was elongated (Figures 1D and S4C), where hypertrophic chondrocytes were rarely observed; instead irregularly organized proliferative chondrocytes were present (Figure 1D, bottom right). These abnormalities were apparently observed after 2 or 3 weeks of age (Figure S4C). In *Slc39a13*-KO chondrocytes, type 10 collagen RNA (*Col10a1*), a marker for hypertrophic chondrocytes, was diminished (Figure 1E, upper). Expression level of *Fgfr3*, *Sox9*, *Sox5* and *Sox6*, all of them crucial for chondrocyte differentiation, were altered in *Slc39a13*-KO chondrocytes (Figure S4B, left). In addition, expression of Indian hedgehog (*Ihh*), a marker for the pre-hypertrophic zone, was downregulated in *Slc39a13*-KO cartilage (Figure 1E, lower). These results strongly suggested that differentiation from proliferative to hypertrophic chondrocytes was impaired in *Slc39a13*-KO mice. The microarray data also revealed that expression of genes regulating cell adhesion and polarity was diminished in *Slc39a13*-KO chondrocytes (Figure S4B, right), suggesting a role of Slc39a13 in cellular organization during chondrocytes differentiation.

### Reduced dentin and alveolar bone, and abnormal craniofacial features in *Slc39a13*-KO mice

**Figure 2:**
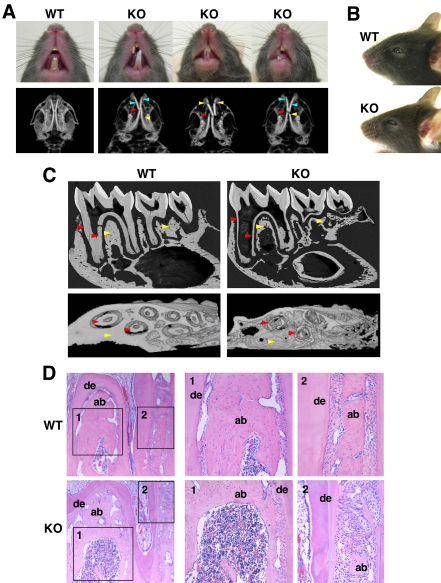
Abnormal teeth and craniofacial development in *Slc39a13*-KO mice. A. *Slc39a13*-KO mice develop abnormal incisor teeth (upper). 5-week-old *Slc39a13*-KO mice show evidence of malocclusion (red arrow head), deformity (blue arrow head), and breakage (yellow arrow head) of incisor teeth. Lower panels show 3D m-CT imaging analysis showing a systemic decrease in bone density and abnormal tooth development of 5-week-old *Slc39a13*-KO mice compared with wild-type littermates. B. Craniofacial features of *Slc39a13*-KO mouse. Eye shows enophthalmos-like appearance and downslanting palpebral fissures in *Slc39a13*-KO mouse. Representative face images of 5-week-old wild-type and *Slc39a13*-KO mouse. C. Root dentin formation of molar teeth (red arrow head) and the bone volume fraction of mandible (yellow arrow head) are remarkably reduced in *Slc39a13*-KO mice. BEN (upper) and 3D m-CT images (lower) of mandibular molar regions in 5-week-old wild-type and *Slc39a13*-KO mice are shown. D. Root dentin and alveolar bone are reduced in *Slc39a13*-KO mice. Sagittal sections of mandibular molar regions in 5-week-old wild-type and *Slc39a13*-KO mice were stained with H&E. Boxed areas are reproduced at higher magnification. de: dentin, ab: alveolar bone.

*Slc39a13*-KO mice showed abnormal incisor teeth (Figure 2A): malocclusion, deformity, and breakage (3D-mCT, Figure 2A, lower). They showed reduced root dentine formation of molar teeth and bone volume of both mandible and alveolar (backscattered electron (BEN) image, Figure 2C upper; 3D m-CT, Figure 2C lower; H&E, Figure 2D), with little morphological change in teeth crowns of molar (Figure 2C, upper right). These data indicated an indispensable role of Slc39a13 in the proper development of root dentin, mandible, and alveolar bones. We also noted some changes in the craniofacial morphology of *Slc39a13*-KO mice. The eyes were sunken, giving an enophthalmos-like appearance, and the palpebral fissures were downslanting (Figure 2B). Measurements of maxilla and mandible bones showed that both bones were remarkably smaller in *Slc39a13*-KO than in wild-type mice (Figure S5). These results indicated that *Slc39a13* has a crucial role for teeth and craniofacial development.

### Decreased dermal and corneal stromal collagen in *Slc39a13*-KO mice

**Figure 3:**
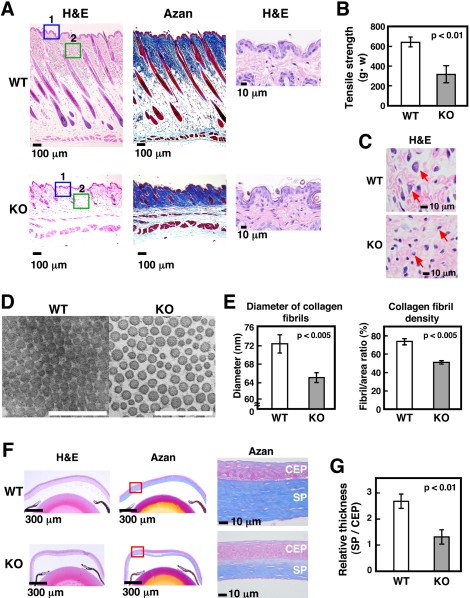
Decreased dermal and corneal stromal collagen in *Slc39a13*-KO mice. A. Dermal collagen is decreased in *Slc39a13*-KO mice. H&E staining shows 5-week-old *Slc39a13*-KO skin is thinner than wild-type littermates (left), without significant difference in epidermis (right; enlarged view of blue-boxed area 1). Azan staining shows collagen fibril is decreased in *Slc39a13*-KO skin (middle). B. Fragility of skin is increased in *Slc39a13*-KO mice. The strength of skin under tension is weakened in 5-week-old *Slc39a13*-KO mice compared with wild-type littermates (n = 3 for each). Data represent mean±S.D. C. Magnified image of green-boxed area 2 in Figure 3A shows morphology of dermal fibroblasts (red arrow) are spindle-shaped and often stellate with cytoplasmic extension in wild-type, while they are mostly round to oval in *Slc39a13*-KO mice. D. TEM images of transversely sectioned dermal collagen from 5-week-old mice are shown. Bar; 500 nm. E. Dermal collagen in 5-week-old *Slc39a13*-KO mice are characterized by thinner in size (left), and lower density of collagen fibrils (right) than that in a wild-type mice. Thirty-nine and 168 areas in TEM images of wild-type and *Slc39a13*-KO samples, respectively were assessed. Data represent mean±S.D. F. Collagen of corneal stroma is decreased in *Slc39a13*-KO mice. H&E (left) and Azan (middle) staining show 5-week-old *Slc39a13*-KO cornea is thinner than wild-type littermates. Magnified images of red-boxed area in middle panel show the width of corneal stroma (substantia propia; SP) is decreased in *Slc39a13*-KO cornea, without significant difference in corneal epithelial cells (CEP) (right). G. Relative ratio between SP versus CEP indicates significant reduction of stromal collagen in *Slc39a13*-KO cornea compared with those of wild-type (n = 5 for each). Data represent mean±S.D.

In addition to hard tissue abnormalities, the strength of skin under tension was significantly reduced (Figure 3B). In fact, dermal collagen fibrils layer was obviously thinner in *Slc39a13*-KO than in wild-type mice (Figure 3A, lower panels of left and middle). Ultrastructual analysis by transmission-electron micrography (TEM) of dermal collagen fibrils in *Slc39a13*-KO mice demonstrated significant decrease and wide variation in size compared with those in wild-type mice (Figures 3D and 3E). At the same time, the epidermis layer did not show significant differences between *Slc39a13*-KO and wild-type mice (Figure 3A, right; enlarged view of blue-box area 1), indicating that the function of collagen-producing cells might be impaired in *Slc39a13*-KO mice. In fact, morphology of *Slc39a13*-KO fibroblasts was changed in dermis (Figure 3C, enlarged view of green-box area 2 in Figure 3A); spindle-shaped and often stellate with cytoplasmic extension in wild-type mice, they were mostly round–shaped in *Slc39a13*-KO mice. In addition to dermis, substantia propria (SP) was markedly thinner in *Slc39a13*-KO eye, while corneal epithelial cell (CEP) layer was not affected (Figures 3F and 3G). Therefore, Slc39a13 may participate in skin and eye development by controlling dermal and corneal stromal fibroblast functions.

### Mutation of *SLC39A13* in Ehlers-Danlos syndrome with short stature and skeletal and connective tissue anomalies

**Figure 4:**
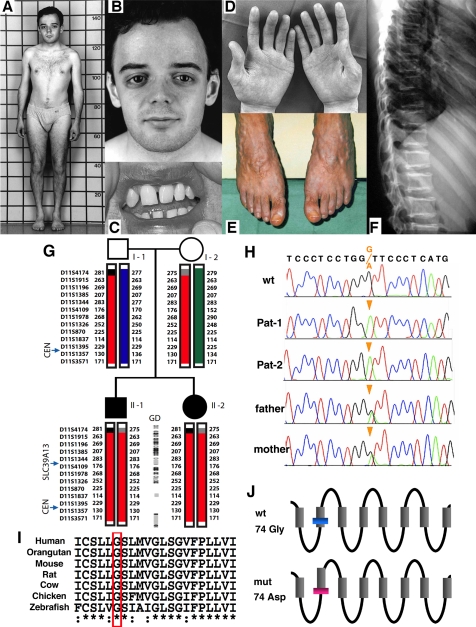
Clinical features, and genetic and molecular evidence of the *SLC39A13* mutation in the two subjects with short stature and EDS. The elder affected sib is shown at age 22 with short stature and with mildly shortened trunk (A), antimongoloid eye slant with lack of periorbital tissue (B), missing upper lateral incisor tooth (C), thin and finely wrinkled skin on the palm of his hands (D), severe varicosity of his lower legs and feet (E), vertebral flattening with sclerosis of the vertebral endplates (F, radiograph taken at age 18 years). G. Autozygous haplotype blocks detected by SNP genotyping are represented by the black boxes, while blocks detected by microsatellite analyses are represented as solid boxes in different colors (the common ancestral haplotype being represented in red). The microsatellites used are shown on the left, and the numbers refer to microsatellites' alleles. Gene density (GD) for local transcripts is shown in the center. Individual genes are depicted as small circles, shaded (for the olfactory receptor genes clusters) or open (all other genes). The relative position of *SLC39A13* and of the centromere (CEN) are also indicated. H. Sequence tracings from *SLC39A13* amplicons showing homozygosity for a G to A transition changing codon 74 from glycine to aspartic acid in the two affected subjects (Pat-1 and Pat-2), as well as heterozygosity in the parents. I. Alignment of amino acid sequences of SLC39A13 protein showing high conservation of sequences and in particular of glycine-74 (red box). J. Assignment of transmembrane domains of SLC39A13 (shaded boxes) was taken from Uniprot (http://www.uniprot.org). The substitution of glycine-74 with aspartic acid was predicted to cause a six-residue shift of the second transmembrane domain towards the carboxy end by HMMTOP program (http://www.enzim.hu/hmmtop/, lower panel).

We studied a pair of sibs with short stature and skeletal and connective tissue disease that could not be ascribed to any of the known EDS types or other connective tissue disorders known so far (Figures 4A–4F, and Case reports). We first observed reduced urinary excretion of hydroxylated collagen metabolites, but mutation analysis of several collagen 1 and 3 genes as well as procollagen hydroxylase genes failed to show pathogenic mutations (Case reports). Analysis of the SNP microarray data obtained in the family members revealed a single larger region of complete homozygosity pericentromeric to chromosome 11 (10 Mb encompassing 227 genes). Further screening of this region by microsatellite mapping (Figure 4G) suggested that two parents shared alleloidentical haplotypic blocks. The location of *SLC39A13* within the region of homozygosity in combination with the strong analogy between the clinical features in our patients and those observed in the *Slc39a13*-KO mouse designated *SLC39A13* as candidate gene. Mutation analysis showed that both affected individuals were homozygous, and the parents were heterozygous, for a G to A transition at nucleotide c.221 (c.221G>A) predicting the non-conservative amino acid substitution G74D (Figure 4H). UniProt (Universal Protein Resource, http://www.uniprot.org) describes the potential structural topology of SLC39A13/ZIP13 as an eight-transmembrane protein as consistent with other family members (Figure 4J) [34], [35], [36], and glycine −74 is located in the second transmembrane domain of SLC39A13 and conserved through all vertebrate species down to fish (Figures 4I and 4J, and data not shown). Screening of 128 chromosomes of Caucasian descent and an additional 48 chromosomes from Portuguese descent did not reveal any instance of the c.221G>A mutation. Thus, we inferred that c.221G>A/G74D was pathogenic, further establishing the important role of SLC39A13 in connective tissue development in mouse and human.

### The Zn transporter Slc39a13 controls intracellular Zn distribution

**Figure 5:**
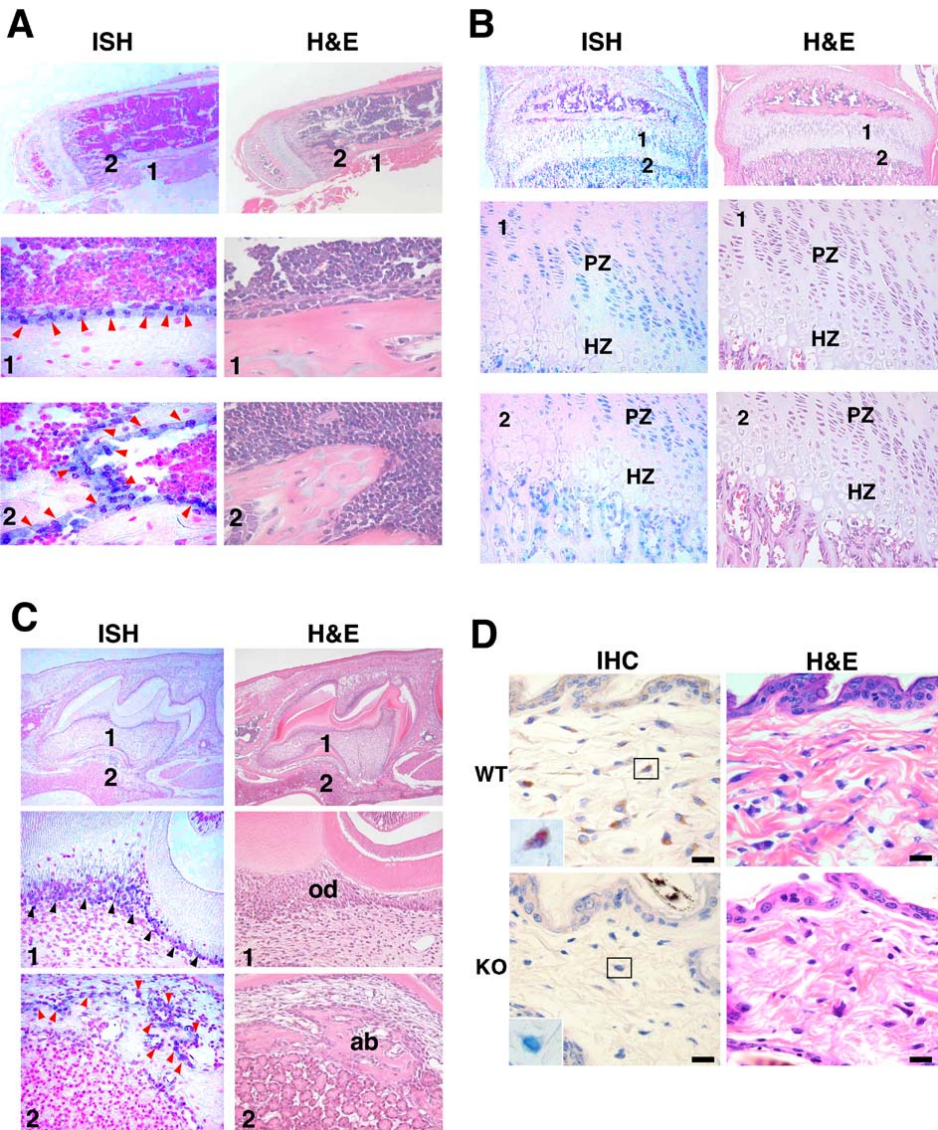
Cellular localization of Slc39a13. A. *Slc39a13* gene is expressed in osteoblasts (1 and 2; red arrow head) in tibia of 4-week-old mice. Regions indicated as 1 and 2 in top are magnified as 400 times. ISH images. B. *Slc39a13* mRNA is expressed in proliferative zone of growth plate (left) at 3-week-old tibia. Region indicated as 1 and 2 in top are enlarged at lower panels. PZ: proliferative zone. HZ: hypertrophic zone. C. *Slc39a13* gene is expressed in odontoblasts of 10-day-old molar teeth. ISH analysis shows *Slc39a13* is expressed in odontoblast (black arrowhead) lining the dentin of crown (1), and in osteoblast (red arrowhead) on the surface of alveolar bone (2). Regions indicated as 1 and 2 in top are magnified as 400 times. od: odontoblasts, ab: alveolar bone. D. Slc39a13 protein expression in dermal fibroblasts. IHC analysis shows Slc39a13 protein is expressed in fibroblasts in dermis of 5-week-old wild-type mice (left upper). Enlarged images of boxed areas are shown in IHC images. Bar indicates 10 µm.

Taken together, all results obtained from *Slc39a13*-KO mice and human cases indicated that Slc39a13 was required for connective tissue development. Indeed, *Slc39a13* gene was relatively highly expressed in some tissues such as bone and eye (Figure S1C). This gene was also expressed in osteoblasts of tibia (Figures 5A1 and 5A2) and of alveolar bone (Figure 5C2), in proliferative zone of growth plate (Figure 5B), and in odontoblasts on the forming of the dentine of crown in molar tooth (Figure 5C1). Fibroblasts in reticular layer of dermis of skin expressed Slc39a13 protein (Figure 5D). Collectively, Slc39a13 was expressed in cells essential for connective tissue development.

**Figure 6:**
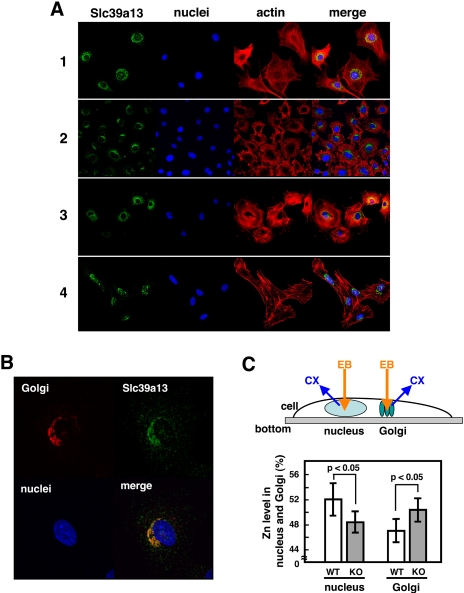
Slc39a13 controls intracellular zinc distribution. A. Slc39a13 protein locates on perinuclear region in primary osteoblasts (1), chondrocytes (2), pulpal cells (3), and dermal fibroblasts (4). Slc39a13, nuclei, and actin were stained with anti-Slc39a13 specific antibody, DAPI, and phalloidin, respectively. Confocal microscopic images. B. Slc39a13 is localized in Golgi. Confocal microscopic analysis using dermal fibroblasts. Golgi, Slc39a13, nuclei, and actin is stained with anti-GM130 antibody, anti-Slc39a13 specific antibody, DAPI, and phalloidin, respectively. C. Upper: Schematic diagram of EPMA to detect intracellular zinc level. Golgi and nucleus in primary dermal fibroblasts are scanned by electron beam (EB), and characteristic X-ray (CX) for zinc is detected. Lower: Slc39a13 involves in control of intracellular zinc distribution. The ratio of zinc level in Golgi or nucleus versus total Zn in those organelles (n = 11 and 10 for wild-type and *Slc39a13*-KO dermal fibroblasts, respectively) was obtained by calculating counts per seconds (cps) of CX for zinc generated by EB scanning; Zn level in each organelle (%) = Each organelle Zn (cps)/{nuclear Zn (cps)+Golgi Zn (cps)}×100. Data represents ±S.D.

Slc39a13 belongs to the SLC39/ZIP family of Zn transporters, and therefore is expected to be located on the cell surface and/or on the lumen of intracellular compartments [37], although neither its cellular localization nor its function has been known previously. We found that Slc39a13 protein was localized in perinuclear region of osteoblasts, chondrocytes, pulpal cells, and of fibroblasts (Figure 6A), and mainly located in Golgi apparatus (Figure 6B), suggesting that Slc39a13 protein functions as an intracellular Zn transporter in connective tissue forming cells. Consistent with this hypothesis, while the overall Zn concentration in serum and in dermal fibroblasts as a whole was similar in wild-type and in *Slc39a13*-KO mice (data not shown), electron probe X-ray micro analysis (EPMA) capable of detecting Zn level in a restricted area of a single cell (Figure 6C, upper) [38] revealed that Zn level in Golgi was increased in *Slc39a13*–KO primary dermal fibroblasts as compared to that in wild-type cells (Figure 6C, lower right). Considering the fact that members of the SLC39s/ZIPs family function as transporters of Zn from the extracellular space into the cytosol [37], all the evidence supports the notion that this transporter Slc39a13 functions as a Zn transporter transporting Zn from the Golgi to the cytosol and thus influences the Zn level at least in areas of the cytosol, adjacent to the Golgi membrane. We were unable to detect a significant change of the average Zn concentration in the cytosol because apparently, the Zn concentration detected by EPMA was very variable (data not shown); cytosol space is broad and Zn content seems to be heterogeneous from area to area in cytosol. We also found that the Zn concentration in the cell nucleus was decreased in the absence of Slc39a13 (Figure 6C, lower left), implicating that Slc39a13 also affects the nuclear translocation of either Zn-binding proteins, or Zn itself, or both. In their complexity, the results indicate that Slc39a13 affects the intracellular distribution of Zn in connective tissue forming cells.

### Slc39a13 is involved in BMP/TGF-β signaling pathways in connective tissue forming cells

**Figure 7:**
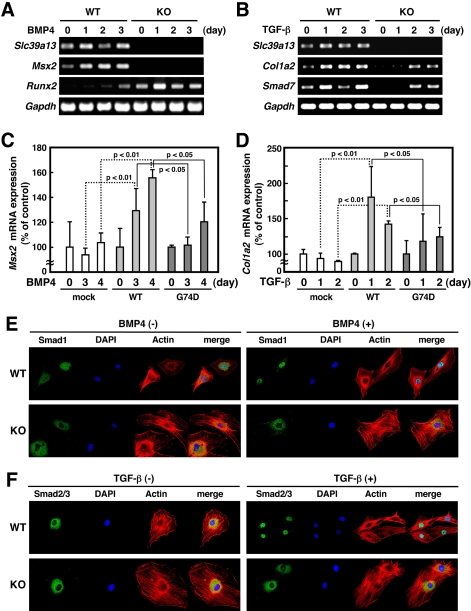
Slc39a13 is involved in BMP and TGF-β -mediated signaling. A. Slc39a13 is involved in BMP4 -mediated gene expression. Primary osteoblasts were stimulated with 50 ng/ml of BMP4 for indicated periods, and gene expression including *Slc39a13*, *Msx2*, *Runx2*, and *Gapdh* was examined by RT-PCR. B. Slc39a13 is involved in TGF-β –mediated gene expression. Primary dermal fibroblasts were incubated with 2.5 ng/ml of TGF-β1 for indicated periods, and gene expression of *Slc39a13*, *Col1a2*, *Smad7*, and *Gapdh* was examined by RT-PCR. C and D. Ectopic expression of mouse wild-type Slc39a13 (WT), but not of G74D mutant (G74D), rescued BMP4 or TGF-β -induced gene expression in *Slc39a13*-KO primary osteoblasts (C) or dermal fibroblast (D), respectively, assessed by real-time PCR. Values represent means±S.D. of three separate experiments. E and F. Slc39a13 is involved in BMP/TGF-β –induced nuclear localization of Smad proteins. Primary osteoblasts (E) or dermal fibroblasts (F) were stimulated with either 50 ng/ml of BMP4 for 15 minutes (E, right panels) or 10 ng/ml of TGF-β1 for 30 minutes (F, right panels), respectively, followed by staining for Smad1, Smad2/3, nuclei (DAPI), and actin (Actin). Confocal microscopic images.

To investigate further the consequences of genetic ablation of Slc39a13, we explored molecular events taking place in *Slc39a13*-KO mice by microarray analysis using primarily isolated osteoblasts and chondrocytes. The pathway-Express program [39] indicated that only BMP/TGF-β signaling was the cascade that was commonly perturbed in both cell types by loss of *Slc39a13* (P-value of hypergeometric distribution with Bonferroni type multiple testing correction, <0.01) (Figure S6A). Consistent with this, we observed changes of gene expression in *Scl39a13*-KO mice, such as depletion of *Msx2*, a homeobox gene crucially involved in BMP-mediated bone and teeth development (Figures S7 left and S8A), abnormal accumulation of *Runx2*, an essential gene for osteoblast maturation (Figure S7, right), and decrease of dermal type 1 collagen (Figure S8B). In addition, BMP4 stimulation in *Slc39a13*-KO primary osteoblasts failed to induce *Msx2* (Figure 7A), and *Runx2* was instead overexpressed (Figure 7A). *Slc39a13*-KO primary dermal fibroblasts showed reduced induction of *type 1 Collagen* RNA (*Col1a2*) and *Smad7* in response to stimulation with TGF-β (Figure 7B). Ectopic expression of Slc39a13 in *Slc39a13*-KO primary osteoblasts and dermal fibroblasts recovered responsiveness against BMP4 and TGF-β (Figures 7C, 7D and S10), establishing an essential role of Slc39a13 for BMP4 and TGF-β signaling. Importantly, mouse Slc39a13 carrying the homologous G to A substitution (G74D, Figures 7C and 7D) as well as human SLC39A13 having the G to A mutation obtained from EDS patients (data not shown) was unable to fully rescue the unresponsiveness of osteoblasts and fibroblasts isolated from *Slc39a13*-KO mice against BMP and TGF-β, respectively, indicating that this mutation causes a loss of function.

Smad transcription factors are major signal transducers of BMP and TGF-β signaling [40], [41]. They are phosphorylated by the activated receptor complex, and subsequently translocate to the nucleus to initiate transcription of their target genes. BMP4 and TGF-β stimulation induced equivalent phosphorylation level of Smad 1/5/8 (Smads) in *Slc39a13*-KO osteoblasts and Smad2 (Smad2) in *Slc39a13*-KO dermal fibroblasts, respectively (Figure S6B). In contrast, nuclear localization of Smad proteins (Figures 7E, 7F and S9C) as well as phosphorylated Smad proteins (Figures S9A, S9B and S9D) was disrupted in BMP4 or TGF-β-stimulated *Slc39a13*-KO cells. This was further confirmed by immunoblotting utilizing subcellular fractions (Figures S9E and S9F). These results suggested that Slc39a13 was involved in nuclear localization of Smad proteins, but not for their phosphorylation, in connective tissue forming cells.

## Discussion

We herein described (1) that SLC39A13 is crucially involved in connective tissue development, (2) the emerging role of the Zn transporter SLC39A13 in BMP/TGF-β signaling in corresponding tissues and cells, (3) that SLC39A13 dysfunction causes skeletal and connective tissue disease both in mouse and man, (4) and thus that *Slc39a13-*KO mouse is a novel animal model for human connective tissue diseases.

*Slc39a13*-KO mice show osteopenia due to the reduction of osteoblast activity (Figures 1C and S3). As shown in Figure 7, loss of Slc39a13 caused dysregulation of BMP/TGF-β -mediated gene expression including expression of *Runx2* and *Msx2*, both of which are critically involved in bone, tooth, and craniofacial skeletogenesis [42], [43], [44], [45], [46]. In BMP4-stimulated *Slc39a13*-KO osteoblasts, we observed simultaneous upregulation and depletion of *Runx2* and *Msx2*, respectively (Figure 7A). This pattern of expression was also observed *in vivo* (Figure S7). Runx2 is essential for osteoblast differentiation [42], meanwhile it represses functional differentiation of osteoblasts when overexpressed in mice [47], suggesting that proper control of its spatio-temporal expression thus seems to be important for osteoblast differentiation *in vivo* [48], [49]. Msx2 is reported to promote osteoblast differentiation independently of Runx2 [50]. Therefore, the Slc39a13 -*Msx2* gene expression axis may also be critical to lead appropriate skeletal and craniofacial development. *Slc39a13*-KO mice show evidence of impaired differentiation of chondrocytes in cartilage: expression of *Fgfr3* and *Sox9* is dysregulated (Figure S4B, left), and that of *Ihh* is disrupted (Figure 1E, lower). The gain-of-function mutant of *Fgfr3* causes achondroplasia [51], *Sox9* controls chondrocyte differentiation via *Sox5* and *Sox6* expression [52], and *Ihh* is a master gene of bone development under BMP control [53], [54], suggesting that Slc39a13 exerts a profound influence on genes crucial to chondrocyte differentiation. We also found irregular formation of chondrocyte columns in *Slc39a13*-KO growth plate (Figure 1D, bottom right), where expression of genes required for tight junction and cellular polarity [55], [56] were diminished (Figure S4B, right). The irregularity in cellular morphology was also observed in *Slc39a13*-KO odontoblasts in the molar tooth (Figure S8A, bottom left) and dermal fibroblasts (Figure 3C); thus, Slc39a13 have a role in growth plate architecture and cellular organization, implicating the possible involvement of Slc39a13 in BMP/TGF-β signalings since they are well recognized to control cell adhesion, polarity, and movement [57], although we cannot exclude the possibility that other signaling pathways are also affected by loss of Slc39a13. In teeth, *Slc39a13*-KO mice show reduced normal formation of root dentin in molar but no significant morphological changes in the molar crown (Figure 2C, upper right), suggesting that teeth development normally initiates without Slc39a13 and proceeds until bell stages. From late bell stage, the epithelial and mesenchymal interactions between ameloblasts and odontoblasts are activated, resulting in mineralization, and the root of a tooth begins its development once the crown formation has been established [58], [59]. In *Slc39a13*-KO mice, odontoblasts cannot sufficiently induce target genes of BMP signaling such as *Msx2* (Figure S8A), thus reduction of the width of root dentin may occur since BMP has a crucial role in this stage [60]. Intriguingly, the root was elongated normally in *Slc39a13*-KO molar tooth (Figure 2C, upper right), suggesting that Slc39a13 is required for root dentin accumulation but not for root elongation. The skin of *Slc39a13*-KO mice was abnormally fragile, most likely because of the reduction in dermal collagen fibrils (Figures 3D and 3E). Similarly, corneal stromal collagen was reduced in *Slc39a13*-KO eyes (Figures 3F and 3G). A functional dysregulation of *Slc39a13*-KO fibroblasts (Figures 3C and 7B) is a plausible reason why both skin and corneal development are impaired in *Slc39a13*-KO mice, since fibroblasts and TGF-β are required for their development and repair [26], [61], [62]. *Slc39a13*-KO mice also showed lipoatrophy; the subcutaneous layer of adipose tissue was significantly thinner in *Slc39a13*-KO than in wild-type mice (Figure 3A, lower left), a finding present also in the EDS patients (Case reports), suggesting that Slc39a13 may determine the fate of other mesenchymal-originated cells like adipocytes. It is also noteworthy that the physical strength of the ocular bulbs in *Slc39a13*-KO mice was reduced (data not shown); loss of Slc39a13 may be pathogenically linked to connective tissue disorders associated with fragile eyes.

These mouse phenotypes of skeletal, dental, dermal, ocular, and craniofacial changes were strongly reminiscent of human EDS and OI [29], [30], [63], [64], [65], [66]. We investigated a pair of sibs who had short stature in combination with skeletal and connective tissue changes that evoked EDS and OI. The *SLC39A13* gene was contained in a single region of homozygosity on chromosome 11, and mutation analysis revealed a point mutation predicting the substitution of a highly conserved amino acid (G74D) in the second transmembrane domain (Figures 4I and 4J). A similar substitution of the corresponding glycine (G330) to aspartic acid (G330D) in the first transmembrane domain of SLC39A4/ZIP4 has been associated with the genetic disease, AE[8]. We showed that the homologous mutation in SLC39A13 indeed causes loss of function (Figures 7C and 7D). Accordingly, the individuals homozygous for the *SLC39A13* loss of function mutation were affected by a disorder analogous to that observed in the *Slc39a13*-KO mice and corresponding to the novel EDS variant. Indeed, the recapitulation of the mouse phenotype by the human disorder is extensive, and includes osteopenia, short stature, thin and fragile skin, enophthalmos, thin sclerae, downslanting palpebral fissures and dental anomalies. In agreement with our data, and while our manuscript was being prepared, Giunta et al reported two families with a *SLC39A13* mutation and a novel type of EDS [31]. This mutation they found was different from the one we identified but was also predicted to cause loss of function. These authors focused on the reduced excretion of hydroxylysine metabolites and suggest inhibition of lysyl hydroxylase as the probable pathogenetic cause. Indeed, we had also observed reduced urinary excretion of hydroxylated collagen metabolites in our subjects. However, the overall clinical picture of our patients and those described by Giunta et al. is different from that of lysyl hydroxylase deficiency (EDS VIA) with normal stature but severe muscular hypotonia and marked looseness of skin and joints; thus, it is clear that the pathogenetic mechanism postulated by Giunta et al., i.e., secondly inhibition of procollagen lysyl hydroxylase activities, may be operational but does not account for the short stature and the additional features, including hypodontia, observed both in the patients and in the mouse model. The defect in BMP/TGF-β signaling that we have described is more consistent with the phenotype; impaired TGF-β signaling fits well with short stature in mouse and human, as increased TGF-β activity has been regarded as the cause of tall stature in Marfan syndrome, another inherited disorder of connective tissue [67], [68]. Notably enough, some clinical spectrum of the EDS patients and of *Slc39a13*-KO mice actually resemble that of human cases and animal models of Zn-deficiency, that show developmental abnormalities including dwarfism, bone growth retardation, and increase of skin fragility [16], [17], [22]. Thus, our combined findings in the mouse model and in the human subjects are unequivocable and point to a “Zn - connective tissues - BMP/TGF-β signaling” connection that deserves thorough exploration.

Our data showed Slc39a13 is involved in BMP/TGF-β signaling pathways in connective tissue forming cells and in nuclear translocation of Smad proteins (Figures 7E, 7F, and S9). Smad proteins are phosphorylated downstream of BMP or TGF-β receptor complex, followed by nuclear translocation. Among the Smad proteins, all receptor-regulated Smad (R- Smad) and Smad4 possess a Zn-binding motif in the MH1 domain for their DNA binding [69]. At the moment, we do not know how Slc39a13 regulates the nuclear localization of Smad proteins, but our results show that the Zn transporter Slc39a13 affects either directly or indirectly BMP and TGF-β signaling pathways. Considering the fact that there was no change of serum Zn concentration in both cases of human patients (Case reports) and of *Slc3913*-KO mice (data not shown) and that Slc39a13 is located in Golgi (Figure 6B), it seems likely that Slc39a13 regulates the intracellular Zn distribution. In fact, the Zn concentration at the whole-cell level was unchanged, but the Zn level in the Golgi was increased in *Slc39a13*-KO cells (Figure 6C, lower right), indicating that Slc39a13 functions as a Zn transporter allowing for efflux of Zn from the Golgi into the cytoplasm, where Zn is expected to interact with Smad. Our data supported that Slc39a13 functions as a Zn transporter controlling intracellular Zn distribution, although we cannot exclude the possibility that Slc39a13 may transport other metals, like Slc39a2/Zip2 for iron and calcium [70], Slc39a14/Zip14 for iron [71], and Slc39a8/Zip8 for cadmium [72]. At the moment it remains unknown how Zn is transferred to its target molecule. It may be simplistic to assume that Zn transporters such as Slc39a13 simply shuttle Zn ions across the Golgi membrane, and that their effect is the result of changes in the concentration of Zn in one or the other cell compartment. Or, unknown “metal chaperone(s)” may transfer Zn directly from a given Zn transporter to Zn binding molecule [73]. The mechanisms how Slc39a13 regulates the nuclear localization of Smad proteins should be an intriguing novel question, and the demonstration of a complex perturbation of intracellular signaling and of deregulation of gene expression in Slc39a13-ablated tissues and cells indicates avenues for further research.

Taken together, the results obtained in *Slc39a13*-KO mice and patients with EDS establish that the Zn transporter SLC39A13 controls intracellular Zn distribution and is involved in connective tissue development. We showed SLC39A13 is required for full activation of BMP and TGF-β signaling through controlling the intracellular localization of Smad in connective tissue forming cells. This would be at least one of the possible reasons why linear growth, bone mineralization, root dentin formation, and fibroblastic collagen synthesis are impaired in *Slc39a13*-KO mice and the EDS patients. The *Slc39a13*-KO phenotype, however, seems to differ from typical BMP/TGF-β related-KO mice which show more severe phenotypes than *Slc39a13*-KO mice [74]. This could be explained in the following ways: 1) In *Slc39a13*-KO, BMP/TGF-β signaling is affected only in those tissues where *Slc39a13* is expressed and functions non-redundantly. 2) In BMP or TGF-β related-KO mice, all tissues are defective of their signaling. 3) In *Slc39a13-*KO mice, in addition to BMP/TGF-β signaling, other signaling pathways might be affected; all of these issues should be unfolded in the future. Our study yields new insights into the relevance of Zn transporters in the biological events, and illustrates their role in health and disease. Thus, further exploration of the role(s) of Zn transporters and of their dysfunction will reveal the role(s) of Zn as a signaling molecule, shed light on other genetic disorders of connective tissue, and help to understand the pathophysiology of Zn-deficiency.

## Materials and Methods

### Generation of *Slc39a13* -KO mice

Generation of knockout mouse line of the *Slc39a13* gene was performed using previously described methods [75]. A bacterial artificial chromosome (BAC) clone containing the mouse *Slc39a13* was screened from an embryonic stem (ES) cell–BAC library [76]. A targeting vector was created to eliminate the genomic region encompassing exons 6–8 by inserting a *Neo*-cassette into a region between exons 5 and 9 of *Slc39a13* and deleting the intervening exons (Figure S1A). This region contains the conserved HEXPHEXGD motif common to SLC39/ZIP family members [2]. This vector was introduced into R1 ES cells and cloned homologous recombinants were selected with antibiotics and the genotypes verified. We developed chimeric mice with the targeted ES cell clones and obtained homozygous mice by interbreeding the offspring. Heterozygous mice were phenotypically normal, and crosses between heterozygotes produced homozygous mutant mice according to Mendelian expectations. All procedures including mouse handling were conducted according to guidelines approved by the RIKEN Institutional Animal Care and Use Committee. Genotyping was done by PCR using LA taq (TAKARA BIO Inc.) (Figure S1B). Primers used for genotyping are;

F; 5′-CTTGTAGCCCACCAATCAGAGACCGATGAT-3′
R1; 5′-TGGTGGTCAGAAGCCCGATCT-3′
R2; 5′-GCTGCTAAAGCGCATGCTCCAGACTGCCTT-3′

### X-ray analysis of bones and teeth, and bone histomorphometry

Radiographs, BMD, measurement of long bone length, and 3D-mCT scans of bone were taken using a composite X-ray analyzing system (In vivo 3D Micro X-ray CT System R_mCT, Rigaku). The dissected mandibles were scanned using micro CT (MCT-100 MFZ, HITACHI). BEN was scanned using Electron Probe X-ray Micro Analyzer (JXA9200 II, JOEL). Bone histomorphometric analysis was performed by using the left tibia from 4-week-old mice (n = 5) injected with calcein (Wako Pure Chemical Industries, Ltd.) for *in vivo* fluorescent labeling (16 mg/kg body weight/10 ml) at 1 and 4 days before sacrifice. The tibia was fixed in 70% ethanol, and the undercalcified bone were embedded in Glycolmetacrylate. 3-micrometer-thick section was cut longitudinally in the proximal region of the tibia, and stained for toluidine-blue-O and tartrate-resistant acid phosphatase (TRAP). Histomorphometry was performed with the semiautomatic image analyzing system (Osteoplan II; Carl Zeiss) linked to a light microscope. The histomorphometric measurements were made at 400 times using a minimum of 17∼20 optical fields in the secondary spongiosa area from the growth plate-metaphyseal junction. Nomenclature, symbols, and units used are those recommended by the Nomenclature Committee of the American Society for Bone and Mineral Research [77].

### Skeletal and teeth histology

Paraffin sections (4∼6 µm) processed from tibia of 1, 2, 3 and 4-week-old mice, mandible of 5-week-old mice, and molar tooth of 10 day-old mice were prepared for H&E staining, and for *in situ* hybridization (ISH). Digoxigenin-labeled antisense RNA probes for *Slc39a13*, *Msx2*, *Runx2*, *Col10a1*, and *Ihh* were used for ISH by the method of GENOSTAFF Inc. The sections used in ISH analysis were counterstained with Kernechtrot stain solution. Probe sequences and hybridization conditions are available upon request.

### Skin and eye histomorphometry

Skin and eye isolated from 5-week-old mice were formalin-fixed and paraffin-embedded. Paraffin sections (4 µm) were cut and stained with H&E and Azan for histological analysis, and also used for immuno histochemistry (IHC). We made a rabbit polyclonal antibody specific to a peptide corresponding to the CLAQPAAEPGLRAVVRNL sequence of mouse Slc39a13, which was used for IHC (Figure 5D) and for confocal microscopic analysis (Figure 6). We confirmed that this antibody was able to detect and was specific to Slc39a13 since it did not show positive signals in *Slc39a13*-KO cells, as shown in Figure 5D. For collagen type I staining, anti-rabbit polyclonal antibody against collagen I (Abcam) was used. To measure the fragility of skin, dorsal skin of 5-week-old mice were chopped in 2×3 cm^2^, and the strength of skin under tension was measured by a spring scale. For transmission electron microscopy, skin biopsy samples were taken from dorsal skin of 5-week-old wild-type and *Slc39a13*-KO mice, and prepared as previously described [78]. For measurement of collagen fibril density, the superficial dermis was photographed in the central portion, and diameters were measured along the minor axis of cross-sections using Image-Pro Plus (Media Cybernetics). Quantitation of fibrils/nm^2^ was also measured from transmission electron micrographs of cross-sections from dermis of the dorsal skin region of mice using Image-Pro Plus (Media Cybernetics).

### Confocal microscopy and immunoblotting

Cells cultured on glass coverslips in 35-mm Glass base dishes (Iwaki) were stimulated and then fixed with 4% paraformaldehyde in PBS. Immunostaining was done after cells were made permeable with BD Perm/Wash Buffer containing antibodies and 1% BSA. Fluorescence was detected with an inverted spectral Confocal Scanning system TCS SP2 AOBS (Leica) with an oil immersion 63× objective. Images were processed with Adobe Photoshop Version 7.0. GM130 antibody (clone35, BD Transduction Laboratories), DAPI (Molecular Probes), and Alexa Fluor®546 phalloidin (Molecular Probes) were used to visualize Golgi, nuclei, and actin, respectively. Alexa Fluor® 568 F(ab')2 fragment of goat anti-mouse IgG (Molecular Probes), Alexa Fluor® 488 F(ab’)2 fragment of goat anti-rabbit IgG (Molecular Probes) were used for secondary staining. The following antibodies were used for confocal microscopy and immunoblotting; anti-Smad1 (Zymed), anti-Smad2/3 (BD Transduction Laboratories), anti-phosphorylated Smad1/5/8 (Cell Signaling), and anti-phosphorylated Smad2 (Cell Signaling). Anti-tubulin (B-5-1-2, Sigma), anti-Flag (Sigma), anti-HDAC1 (Sigma). We made a rabbit antibody by immunization with a peptide corresponding to the NSKEDPSQAPSKDPTAA sequence of mouse Slc39a13, which was used for immunoblotting (Figure S6). As shown in Figure S6B, this antibody reacted with a 40 kDa molecule in wild type cells but not in *Slc39a13*-KO cells. BMP4 and TGF-β1 were purchased from R&D systems.

### Subcellular fractionation

Cells were stimulated with BMP4 or TGF-β1 for indicated periods, and then were lysed with hypotonic buffer (20 mM HEPES pH 7.9, 0.1 mM EDTA, 0.2% NP-40, 10 mM NaCl, 1 mM DTT) supplemented with proteinase inhibitors for 5 min. After centrifugation at 10,000×g for 1 min, cytoplasmic fraction was isolated as supernatant. Precipitates were then incubated with extraction buffer (20 mM HEPES pH 7.9, 0.1 mM EDTA, 0.2% NP-40, 420 mM NaCl, 1 mM DTT) supplemented with proteinase inhibitors at 4°C for 30 min. Nuclear fraction was subsequently extracted by centrifugation (10,000×g, 4°C, 10 min). For total cell lysates, cells were suspended with lysis buffer (1% NP-40, 20 mM Tris-HCl pH 7.4, 150 mM NaCl) supplemented with proteinase inhibitors, and lysates were cleared by centrifugation (10,000×g, 4°C, 10 min). 20 µg of each fraction were subjected to SDS-PAGE, followed by immunoblotting.

### Measurement of zinc distribution in cell

Electron Probe X-ray Micro Analyzer (JXA9200 II, JOEL) assessed intracellular zinc distribution in primary dermal fibroblasts. Wild-type and *Slc39a13*-KO dermal fibroblasts were cultured in 6 well plates, and cells were fixed with 2.5% glutaraldehyde in 0.1 M sodium cacodylate buffer and post-fixed in 1% osmium tetroxide. Subsequently, the cells were dehydrated in an ascending series of alcohol and embedded in epoxy resin. Electron beam scanned Golgi and nucleus of embedded cells. Characteristic X-ray for zinc generated by electron beam exposure was detected.

### Gene expression profiles

Gene expression profiling was conducted using total RNAs obtained from primary osteoblasts and chondrocytes of wild-type and *Slc39a13*-KO mice. Biotinylated cRNA was synthesized using a One-Cycle Target Labeling kit (Affymetrix) according to the manufacturer's manual and hybridized with Mouse Genome 430 2.0 Arrays (Affymetrix). The signal intensities for each probe set were calculated using a gcRMA method in a Genespring GX 7.3 software package (Agilent). Using lists of genes exhibiting 2-fold changes between wild-type and *Slc39a13*-KO cells, pathway analysis was conducted on a Pathway Express program (http://vortex.cs.wayne.edu/projects.htm#pathways-Express). The raw data for microarray analysis are available from the Gene Expression Omnibus (GEO) of the National Center for Biotechnology Information (NCBI; series accession nos. GSE10555 and GSE10556). For RT-PCR analysis, total RNA was extracted from cells and several tissues of C57/B6J mice using Sepasol-RNA I (Nacalai tesque), and reverse-transcribed with oligo-(dT) primer and a reverse transcriptase (ReverTra Ace, TOYOBO). SYBR Green PCR Master Mix (Applied Biosystems) was used for real-time PCR analysis. Primer sequences for RT-PCR and real-time PCR are listed in Supplemental Tables S2 and S3, respectively.

### Statistics analysis

Differences among multiple groups were compared by 1-way ANOVA followed by a post-hoc comparison using Fisher's PLSD test. The two-tailed Student's test was used to analyze difference between 2 groups.

### Human studies

The committee approved the studies and confirmed that informed consent was obtained from human subjects as follows: The patients were not recruited in a prospective study, but came to us with a request for diagnostic help and genetic counseling owing to our reputation as medical experts in skeletal and connective tissue diseases. We have seen them at the Lausanne University Hospital. Thus, our relationship with them falls under the physician-patient relationship as defined by Swiss law; every single procedure (clinical examination, venipuncture, radiography and skin biopsy) is directly related to the diagnostic procedure and is not per se a research procedure. Equally, analysis of the patients' biologic material (urine, blood, DNA, cell culture) is part of the routine diagnostic procedure to confirm or rule our known genetic entities (in our case, the known forms of Ehlers-Danlos syndromes). Although written consent is required only for surgical operation with anaesthesia, which was not performed in our case, we obtained both oral and written informed consent. The IRB has granted approval for the informed consent for research procedures of this type (i.e., non-surgical) to all activities performed within the Swiss National Foundation research project “Molecular Bases of Human Chondrodysplasias” (PIs: A. Superti-Furga and L. Bonafé). This is to cover for the performance of additional procedures such as the sequencing of “novel” genes and the study of cell cultures in such patients. The patients themselves (two well-informed young adults) have enthusiastically (and thankfully) given oral and written informed consent to study their DNA and cells hoping for results that could help in therapy and/or genetic counseling.

### Case reports

#### Clinical characterization of a novel type of Ehlers-Danlos syndrome

We identified a sib pair, a male and a female, with short stature and a combination of skeletal and connective tissue findings that could not be classified in existing nosologic categories. Briefly, these individuals were born at term from uncomplicated pregnancies. The parents were not knowingly related but originated from the same region in Portugal; they were healthy and of average stature. Both sibs were of normal size and weight at birth but showed progressive short stature beginning in the second half of the first year of life. Among the clinical signs at that time were muscular hypotonia and soft skin, leading to the diagnostic suspicion of the Ehlers-Danlos Syndrome (EDS). During childhood, the main clinical signs and features were thin, fragile, but not hyperelastic skin that bruised easily and was particularly thin on the hands and feet; varicose veins; moderate joint laxity; blueish or greyish sclerae; downslanting palpebral fissures; and hypodontia of one or a few teeth in permanent dentition. Both had astigmatism in childhood. Radiographic examination revealed moderate osteopenia, flattened or biconcave vertebral bodies with flaky irregularity of the endplates as well as mild dysplastic changes at the metaphyses of long bones and of the phalanges. As adults, the affected male and female subjects were, respectively, 145 and 135 cm tall; their body proportions were normal, indicating that the platyspondyly was accompanied by shortening of the long bones. Their skin remained thin and fragile, and the subcutaneous fat tissue was sparse. Both individuals had marked venous varicosities on their feet and legs. The male subject suffered from a cerebral hemorrhage posteriorly to the left putamen at age 21 years, from which he recovered completely. Intellectual development was above average, and there was no history of susceptibility to infections. Thus, the patients had features of the EDS, in particular of EDS type IV (thin and fragile skin, varicose veins, cerebral hemorrhage) and some signs of osteogenesis imperfecta (flattened or biconcave vertebrae and grayish sclerae), but the degree of short stature was unusual, and the facial appearance with downslanting palpebral fissures as well as the dysplastic vertebral bodies similarly did not fit either diagnosis well [29], [30], [79].

#### Biochemical characterization of the novel type of Ehlers-Danlos syndrome

Routine analysis of collagen synthesis in cultured fibroblasts was normal, ruling out a diagnosis of EDS IV and making OI unlikely. Analysis of the urinary excretion of collagen crosslinks revealed that both individuals excreted a reduced ratio of hydroxylated vs. unhydroxylated collagen crosslink products in their urine (Supplemental Table S1). The reduction was statistically significant when compared to that of either parent, but was milder than that observed in lysyl hydroxylase deficiency (EDS type VIA)[80], [81]. A therapeutic trial of vitamin C administered orally at a dose of 1 g/day for one month to stimulate procollagen lysyl hydroxylation in the two affected individuals did not change the urinary excretion pattern. After elucidation of the SLC39A13 mutations, Zn levels were determined twice in both affected individuals in plasma and in urine to test for a possible perturbation of whole-body Zn homeostasis; the Zn levels were within the normal range for adults. This may be consistent with the finding that mouse Slc39a13 is located in the Golgi and therefore SLC39A13 likely regulates the intracellular Zn distribution.

#### Linkage and mutation analysis of the family with the novel type of Ehlers-Danlos syndrome

Based on the slightly but consistently reduced excretion of hydroxylated collagen crosslink products, we first hypothesized that this condition was either a variant of EDS VIA or another enzymatic deficiency leading to impaired procollagen lysyl hydroxylation, but extensive linkage analysis under a model of recessive inheritance and targeted mutation analysis excluded the three procollagen lysyl hydroxylase genes *PLOD1*, *PLOD2*, and *PLOD3* as responsible for the disease; further studies excluded the genes, *COL1A1*, *COL1A2*, *COL3A1*, and *CRTAP*.

### Supporting information

Information for plasmid construction and transfection, skeletal staining, primary cell culture condition, measurement of maxilla and mandibles, and analysis of human biological samples was described in Text S1.

## Supporting Information

Text S1 (0.68 KB DOC MB )

Figure S1 Generation of Slc39a13-KO mice. A. Schematic diagram of the construct used to generate Slc39a13-KO mice. A targeting vector was constructed with a Neo-cassette inserted into the region between exons 5 and 9 of Slc39a13 locus. TK: thymidine kinase B. Infants produced by crosses between heterozygotes were genotyped by PCR using specific primers (F, R1 and R2, shown in Figure S1A). WT: wild-type, HE: heterozygote, KO: Slc39a13-KO. C. Slc39a13 gene expression in mouse tissues assessed by RT-PCR. sLN: superficial inguinal lymph node, mLN: mesenteric lymph node, BM: bone marrow cells, ΔBM: without bone marrow cells.
(0.73 MB TIF)

Figure S2 Growth retardation of Slc39a13-KO mice. A. Left: Little significant difference in skeletogenesis and body size at newborn between wild-type and Slc39a13-KO mice. Bar indicates 1 cm; Right: Body weight of newborn mice (n = 5 for each). Data represent mean±S.D. B. Significant delayed growth is observed after 3 weeks of age (n = 5; male mice). Data represent mean±S.D. C. Both male and female Slc39a13-KO mice show growth retardation (n = 10 for each). Data represent mean±S.E.M. WT: wild-type mice, HE: heterozygote mice, KO: Slc39a13-KO mice.
(1.41 MB TIF)

Figure S3 Skeletal histomorphometry of Slc39a13-KO mice. A-D: Bone histomorphometric analysis. Data represent mean±S.D. A. BMD (bone mineral density) in skull, mandible, cortex, and cancellous zone of femur are decreased in 5-week-old Slc39a13-KO mice compared with wild-type mice (n = 5 for each). B. Bone volume and osteoid thickness of 4-week-old Slc39a13-KO mice are lower than those of wild-type (n = 5 for each). C. Osteoblast function is significantly decreased in Slc39a13-KO mice. A double-labeling analysis of calcein (left), mineral apposition ratio (middle), and first and second calcein bone formation rate (right) of 4-week-old mice (n = 5 for each). D. Osteoclast activity of Slc39a13-KO mice is equivalent to wild-type littermates. Eroded surface (left), osteoclast number (middle), and osteoclast-covered bone surface (right) of 4-week-old Slc39a13-KO and wild-type mice are shown (n = 5 for each).
(0.94 MB TIF)

Figure S4 Abnormal cartilage development in Slc39a13-KO mice. A. Length of femur (upper) and tibia (lower) of 4-week-old Slc39a13-KO mice are shorter than those of wild-type (n = 5 for each). Data represent mean±S.D. B. Genes involved in chondrocyte differentiation (left) and in cell adhesion or polarity (right) are dysregulated in Slc39a13-KO primary chondrocytes. Gene expression profiling by DNA microarray analysis was carried out using total RNA from Slc39a13-KO or wild-type primary chondrocytes. Each gene was normalized to the median of the measurement for that gene. C. Abnormal morphology of growth plate in Slc39a13-KO mice. H&E staining images are shown. PZ: proliferative zone. HZ: hypertrophic zone. Bar indicates 100 µm.
(4.46 MB TIF

Figure S5 Impaired craniofacial skeletogenesis in Slc39a13-KO mice. A. The following points are traced on the cephalometric radiographs: Upper; Ba, Basion, defined as the most posterior-inferior cephalometric maxilla point; Rh, Rhinion, the most anterior point of the nasal bone; and Na, Nasion, the cephalometric point between the nasal bone and the frontal bone. Lower; Me, Menton, the lowest point of the chin; Go, Gonion, the most posterior inferior point at the angle of the mandible; and Co, Condylion, the most posterior superior point on the condyle of the mandible. B-F. Dwarfed craniofacial formation in Slc39a13-KO mice. Analysis of the 5-week-old maxilla and mandible bone reveals that Slc39a13-KO mice are characterized by shorter craniofacial depth (B. Ba-Rh), shorter nasomaxillary depth (C. Na-Rh), shorter cranial base depth (D. Na-Ba), shorter mandibular depth (E. Me-Go), and shorter craniofacial depth (F. Me-Co), compared with wild-type mice (n = 5 for each). Data represent mean±S.D.
(0.75 MB TIF)

Figure S6 Perturbation of BMP/TGF-β signal transduction without affecting phosphorylation of Smad proteins in Slc39a13-KO cells. A. DNA microarray analysis using RNA of primary osteoblasts (left) and chondrocytes (right). Each gene was normalized to the median of the measurement for that gene. B. Smad proteins are normally phosphorylated in Slc39a13-KO cells. Primary osteoblasts or dermal fibroblasts were stimulated with either 50 ng/ml of BMP4 (left), or 10 ng/ml of TGF-β1 (right), respectively for indicated periods. Total cell lysates were separated by SDS-PAGE, followed by immunoblotting with either anti-phosphorylated Smad1/5/8 (pSmads), anti-Smad1, anti-phosphorylated Smad2 (pSmad2), anti-Smad2/3, or anti-Scl39a13 specific antibodies. Anti- α -tubulin antibody was used for control blotting.
(0.84 MB TIF)

Figure S7 Dysregulated expression of tibial Msx2 and Rnux2 in Slc39a13-KO mouse. ISH analysis shows Runx2 is accumulated (right), while Msx2 is diminished (left) in 4-week-old Slc39a13-KO tibia. Regions indicated as 1 and 2 in upper are enlarged as 200 times at middle and lower panels.
(8.41 MB TIF)

Figure S8 Dysregulated expression of molar Msx2 and dermal type 1 collagen expression in Slc39a13-KO mouse. A. ISH analysis shows Msx2 gene expression is diminished in odontoblasts (od) lining the dentin of crown (★) of 10-day-old Slc39a13-KO molar teeth. Regions indicated with ★ in upper are enlarged as 200 times at lower panels. Unorganized odontoblasts are observed in Slc39a13-KO molar. B. Type I collagen level is decreased in Slc39a13-KO skin. Skin section of 5-week-old wild-type and Slc39a13-KO mice were applied for IHC. Bar indicates 100 µm.
(5.71 MB TIF)

Figure S9 Involvement of Slc39a13 in localization of Smad proteins. A and B. Slc39a13 is involved in BMP/TGF-β -induced nuclear localization of phosphorylated Smad proteins. Primary osteoblasts (A) or dermal fibroblasts (B) were stimulated with either 50 ng/ml of BMP4 for 15 minutes (A, right panels) or 10 ng/ml of TGF-β1 for 30 minutes (B, right panels), respectively, followed by staining for phosphorylated Smad1/5/8 (pSmads), phosphorylated Smad2 (pSmad2), nuclei (DAPI), and actin (Actin). Confocal microscopic images are shown. C and D. Ratio of subcellular localization of intact (C) or phosphorylated (D) Smad proteins was obtained by counting cells (n = 50) in confocal microscopy images visualized by anti- Smad1, anti-Smad2/3, anti-phosphorylated Smad1/5/8 (pSmad), or anti- phosphorylated Smad2 (pSmad2) antibodies after BMP4 (left in C and D; for Smad1 and pSmads in osteoblasts) or TGF-β1 (right in C and D; for Smad2/3 and pSmad2 in dermal fibroblasts) stimulation. nuc: nuclear space, cyto: cytoplasmic space, both: both of nuclear and cytoplasmic spaces E and F. Cytoplasmic and nuclear fractions from primary osteoblasts (E) and dermal fibroblasts (F) were separated by SDS-PAGE, followed by immunoblotting with either anti-phosphorylated Smad1/5/8 (pSmads) and anti-Smad1 (E), or anti-phosphorylated Smad2 (pSmad2) and anti-Smad2/3 (F) antibodies. Anti- α-tubulin and HDAC1 antibodies were used for control blotting.
(2.36 MB TIF)

Figure S10 Ectopic expression of Slc39a13. Either empty vector (mock), Flag-tagged wild-type (WT), or G74D mutated (G74D) mouse Slc39a13 expression plasmids were transfected into Slc39a13-KO primary osteoblasts (A) and dermal fibroblasts (B). Their expression level was assessed by RT-PCR (upper), and by immunoblotting using anti-Flag and anti-α-tubulin antibodies at two days after transfection (lower).
(0.72 MB TIF)

Table S1 Molar ratio of hydroxylysyl/lysyl-pyridinoline collagen crosslinks in urine of the two sibs with EDS and in their parents.
(0.71 MB TIF)

Table S2 Primer sequences used for RT-PCR are listed.
(0.97 MB TIF)

Table S3 Primer sequences used for real time-PCR are listed.
(0.77 MB TIF)
