# Correction: The Zinc Transporter SLC39A13/ZIP13 Is Required for Connective Tissue Development; Its Involvement in BMP/TGF-β Signaling Pathways

**DOI:** 10.1371/annotation/a6c35a12-e8eb-43a0-9d00-5078fa6da1bb

**Published:** 2008-11-13

**Authors:** Toshiyuki Fukada, Natacha Civic, Tatsuya Furuichi, Shinji Shimoda, Kenji Mishima, Hiroyuki Higashiyama, Yayoi Idaira, Yoshinobu Asada, Hiroshi Kitamura, Satoru Yamasaki, Shintaro Hojyo, Manabu Nakayama, Osamu Ohara, Haruhiko Koseki, Heloisa G. dos Santos, Luisa Bonafe, Russia Ha-Vinh, Andreas Zankl, Sheila Unger, Marius E. Kraenzlin, Jacques S. Beckmann, Ichiro Saito, Carlo Rivolta, Shiro Ikegawa, Andrea Superti-Furga, Toshio Hirano

The last five authors, Ichiro Saito, Carlo Rivolta, Shiro Ikegawa, Andrea Superti-Furga, and Toshio Hirano, should be noted as joint senior authors on this work.

