# Supplementary material for: The Zinc Transporter SLC39A13/ZIP13 Is Required for Connective Tissue Development; Its Involvement in BMP/TGF-β Signaling Pathways
Source: PLoS One. 2008 Nov 5;3(11):e3642. doi: 10.1371/journal.pone.0003642 (PMC2575416; doi:10.1371/journal.pone.0003642)
Supplement: Text S1 — (0.68 KB DOC MB ) [file pone.0003642.s001.doc]

# Supplemental Methods

**Plasmid construction and transfection**

Full-length mouse *Slc39a13* and *Ihh* cDNA clones were kindly gifted from Dr. Yoshihide Hayashizaki (RIKEN Omics Science Center, Japan) [1]. Mouse *Slc39a13* cDNA was subcloned into p3XFLAG-myc-CMV™-26 expression vector (Sigma). Expression plasmid for mouse *Slc39a13* harboring G74D mutation was created by PCR-based single nucleotide substitution using a following primer set; 5’-GTCCAAGAGGGAGCAGATCCA-3’ and 5’-TGGATCTGCTCCCTCTTGGA-3’. Transfection of expression vector into primary osteoblasts was carried out by electroporation with 350V and 950μF, and into primary fibroblasts by Lipofectamine 2000 (Invitrogen).

### Skeleton staining

Newborn mice were skinned, eviscerated, and fixed in 95% ethanol. They were then defatted for 2 days in acetone and stained sequentially with Alcian blue and Alizarin red S in 2% KOH [2]. The stained skeleton preparations were cleared with 1% KOH and stored in 100% glycerol.

Primary culture of mouse osteoblasts, chondrocytes, pulpal cells, and dermal fibroblasts

For primary osteoblasts, calvaria from newborn mice were dissected aseptically and digested sequentially in isolation buffer (25 mM HEPES pH 7.5, 10 mM NaHCO_3_, 100 mM NaCl, 3 mM K_2_HPO_4_, 1 mM CaCl_2_, 30 mM KCl, 1 mg/ml BSA, 5 mg/ml D-glucose) containing 0.75 mg/ml collagenase and 0.2% dispase. Fractions 3–5 were cultured in α-MEM containing 10% fetal calf serum (FCS) with penicillin and streptomycin. Osteoblasts were stained with ALP (TAKARA BIO INC.). For pulpal cell culture, molar and incisor tooth were isolated from 10-day-old *Slc39a13*-KO and wild-type mice. Pulp were extracted and incubated in 0.4% collagenase in α-MEM for 10 min at 37℃. Pulpal cells were collected and seeded in tissue culture plates with α-MEM containing 10% FCS with penicillin and streptomycin. Primary culture of chondrocytes [3] and dermal fibroblast [4] was performed based on the previous reports. Briefly for chondrocytes, cartilaginous rib cages from newborn mice were incubated in 2 mg/ml collagenase in α-MEM containing for 60 min at 37 ℃, and rinsed with PBS (-) containing penicillin and streptomycin. The rib cages were further incubated in 3 mg/ml collagenase in α-MEM containing for 6 h at 37 ℃, and undigested parts were discarded. Cells were collected and rinsed with α-MEM, passed through cell strainer (BD Falcon), and were grown in α-MEM containing 10% FCS with penicillin and streptomycin. For dermal fibroblasts, skin of 5-week-old mice were isolated after shaving, and washed with PBS (-) containing penicillin and streptomycin. After unwanted tissues were removed, they were chopped into 5 mm square cube. They were washed with PBS (-) containing penicillin and streptomycin, and three pieces were put onφ6cm dish, followed by putting cover glass on them. Dermal fibroblasts were cultured with α-MEM containing 20% FCS with penicillin and streptomycin for 3-5 days, and cover slip and explant were removed when adhesion portion occupied ~50% in the whole area.

### Preparation and measurement of maxilla and mandibles

The heads of 5-week-old *Slc39a13*-KO and wild-type mice were used to isolate maxilla and mandible bones. Cephalometric radiographs were obtained by means of a soft X-ray machine (SOFTEX csm-2, SOFTEX). Preparation and measurement of maxilla and mandibles were performed based on the previous report [5].

### Isolation and analysis of human biological samples

All human samples (urine samples, blood samples, and skin biopsies) were obtained from the two affected individuals as well as from their parents (urine and blood samples) with appropriate informed consent. Genomic DNA was extracted from peripheral blood leukocytes and from fibroblast cultures using routine methods. Genomic DNA from patients and their parents was genotyped by hybridization to the GeneChip Human Mapping 100K microarray (Affymetrix). Individual genotypes were extracted with the CNAT software (Affymetrix) and specifically formatted to undergo analyses with different softwares (Merlin [6], AutoSNPa [7], CNAG [8]). The genetic map for the 100k SNPs was determined by interpolating the SNP map positions from the Rutgers Linkage-Physical Map [9]. Identity by descent of genomic regions was assessed by the use of the “-- ibd" option of the Merlin Package [6]. For mutation analysis of *PLOD2*, *PLOD3*, and COL3A1, we designed primers based on public sequences [10]. In *COL3A1*, we targeted codons relative to lysine residues 263, 284, 860, 977, 1094, and 1106, which are involved in hydroxylation (according to the Swiss-Prot P02461 entry). Additionally, sequences corresponding to the N- and C-terminal non-helical regions (amino acids 149-167 and 1197-1205, respectively) were sequenced. Primers and PCR conditions are available upon request. We also amplified the 9 coding exons of *SLC39A13*, with their flanking intron sequences, and sequenced the PCR products using an ABI Prism 3700 automated sequencer (PE Biosystems). Microsatellite markers chosen to cover the regions of the genes *PLOD1, PLOD2, PLOD3, COL1A1, COL1A2*, and COL3A1 were amplified using commercially available primers. Within the chromosome 11 autozygous region, primers chosen were *D11S4174, D11S1915, D11S1196, D11S1385, D11S1344, D11S4109, D11S1978, D11S1326, D11S870, D11S1837, D11S1395, D11S1357*, and *D11S3571*. PCR primer pairs and PCR conditions (available upon request) were individually tailored to allow optimal amplification of each marker. In all instances, one of the 2 PCR primers was 5' end-labeled with the 6-FAM fluorophore, to allow detection of the amplified amplimers in fluorescent capillary electrophoresis using an Applied Biosystems 3130 DNA Analyzer.

# Isolation of RNA from human primary fibroblasts and gene expression analysis

Primary fibroblasts were cultured from skin biopsies using standard procedures. Fibroblasts were then passaged in T75 flasks and cultured in DMEM with low glucose and L-glutamine (Gibco) containing 10% fetal bovine serum (Gibco) and 50 kU/L penicillin and 50 mg/L streptomycin (mix from Sigma). For RNA isolation, cells at passage 2-3 were trypsinized (with 0.05% trypsin-EDTA, Gibco), and separated from the culture medium by centrifugation. Total RNA was extracted from pelleted cells by the use of Qiashredder columns and the RNeasy kit (Qiagen), including on-column DNase treatment, to obtain final RNA preparations with concentrations ranging from 250 to 450 ng/μl, in RNase-free water. RNA was checked for physical integrity by capillary electrophoresis via the HDA-GT12 System (eGene, Inc.) and for purity by spectrophotometry (ND-1000, NanoDrop). Prior to microarray hybridization, RNA was assayed with an additional step of quality control by the use of the Bioanalyzer 2100 (Agilent). Gene expression was assessed by microarray hybridization to the CodeLink Human Whole Genome Bioarray (GE Healthcare). Fibroblast RNA from the 2 patients and the 4 control anonymous donors was labeled, hybridized and quantified according to previously published procedures [11]. The expression data was RMA (Robust Multi-Array) normalized. The most differentially expressed genes were valued by a Welch t-test. The relevant biological networks implicating differentially expressed genes were assessed with the Ingenuity Systems software.

# Urinary collagen crosslink products

Quantitative analysis of the pyridinium cross-links of collagen, pyridinoline and deoxypyridinoline, in spot urine samples was performed using acid hydrolysis, prepurification by step chromatography, and ion-paired reversed phase high-performance liquid chromatography with fluorescence detection [12,13] using a commercial detection kit (Crosslinks by HPLC, BIO-RAD, Hercules, CA).

1. Okazaki Y, Furuno M, Kasukawa T, Adachi J, Bono H, et al. (2002) Analysis of the mouse transcriptome based on functional annotation of 60,770 full-length cDNAs. Nature 420: 563-573.

2. McLeod MJ (1980) Differential staining of cartilage and bone in whole mouse fetuses by alcian blue and alizarin red S. Teratology 22: 299-301.

3. Lefebvre V, Garofalo S, Zhou G, Metsaranta M, Vuorio E, et al. (1994) Characterization of primary cultures of chondrocytes from type II collagen/beta-galactosidase transgenic mice. Matrix Biol 14: 329-335.

4. Freshney RI (1987) Disaggregation of the tissue and primary culture.; RI F, editor. New York: Alan R Liss. 113-115 p.

5. Kyrkanides S, Kambylafkas P, Miller JH, Tallents RH, Puzas JE (2007) The cranial base in craniofacial development: a gene therapy study. J Dent Res 86: 956-961.

6. Abecasis GR, Cherny SS, Cookson WO, Cardon LR (2002) Merlin--rapid analysis of dense genetic maps using sparse gene flow trees. Nat Genet 30: 97-101.

7. Carr IM, Flintoff KJ, Taylor GR, Markham AF, Bonthron DT (2006) Interactive visual analysis of SNP data for rapid autozygosity mapping in consanguineous families. Hum Mutat 27: 1041-1046.

8. Nannya Y, Sanada M, Nakazaki K, Hosoya N, Wang L, et al. (2005) A robust algorithm for copy number detection using high-density oligonucleotide single nucleotide polymorphism genotyping arrays. Cancer Res 65: 6071-6079.

9. Kong X, Murphy K, Raj T, He C, White PS, et al. (2004) A combined linkage-physical map of the human genome. Am J Hum Genet 75: 1143-1148.

10. Ha-Vinh R, Alanay Y, Bank RA, Campos-Xavier AB, Zankl A, et al. (2004) Phenotypic and molecular characterization of Bruck syndrome (osteogenesis imperfecta with contractures of the large joints) caused by a recessive mutation in PLOD2. Am J Med Genet A 131: 115-120.

11. Rivolta C, McGee TL, Rio Frio T, Jensen RV, Berson EL, et al. (2006) Variation in retinitis pigmentosa-11 (PRPF31 or RP11) gene expression between symptomatic and asymptomatic patients with dominant RP11 mutations. Hum Mutat 27: 644-653.

12. Black D, Duncan A, Robins SP (1988) Quantitative analysis of the pyridinium crosslinks of collagen in urine using ion-paired reversed-phase high-performance liquid chromatography. Anal Biochem 169: 197-203.

13. Al-Hussain H, Zeisberger SM, Huber PR, Giunta C, Steinmann B (2004) Brittle cornea syndrome and its delineation from the kyphoscoliotic type of Ehlers-Danlos syndrome (EDS VI): report on 23 patients and review of the literature. Am J Med Genet A 124: 28-34.
