# Supplementary figures and images for: The Zinc Transporter SLC39A13/ZIP13 Is Required for Connective Tissue Development; Its Involvement in BMP/TGF-β Signaling Pathways
Source: PLoS One. 2008 Nov 5;3(11):e3642. doi: 10.1371/journal.pone.0003642 (PMC2575416; doi:10.1371/journal.pone.0003642)

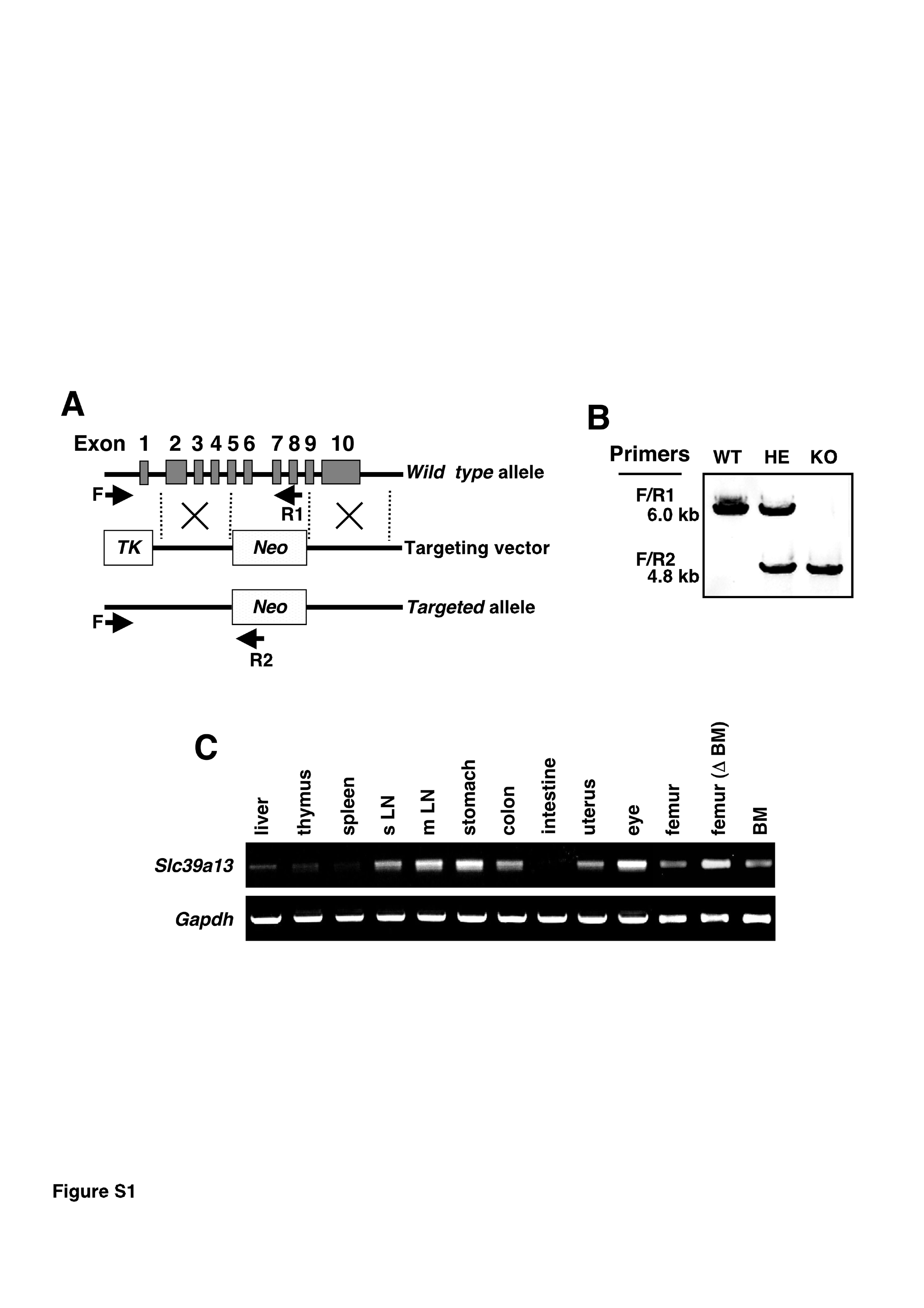

Supplement: Figure S1 — Generation of Slc39a13-KO mice. A. Schematic diagram of the construct used to generate Slc39a13-KO mice. A targeting vector was constructed with a Neo-cassette inserted into the region between exons 5 and 9 of Slc39a13 locus. TK: thymidine kinase B. Infants produced by crosses between heterozygotes were genotyped by PCR using specific primers (F, R1 and R2, shown in Figure S1A). WT: wild-type, HE: heterozygote, KO: Slc39a13-KO. C. Slc39a13 gene expression in mouse tissues assessed by RT-PCR. sLN: superficial inguinal lymph node, mLN: mesenteric lymph node, BM: bone marrow cells, ΔBM: without bone marrow cells. (0.73 MB TIF) [file pone.0003642.s002.tif]

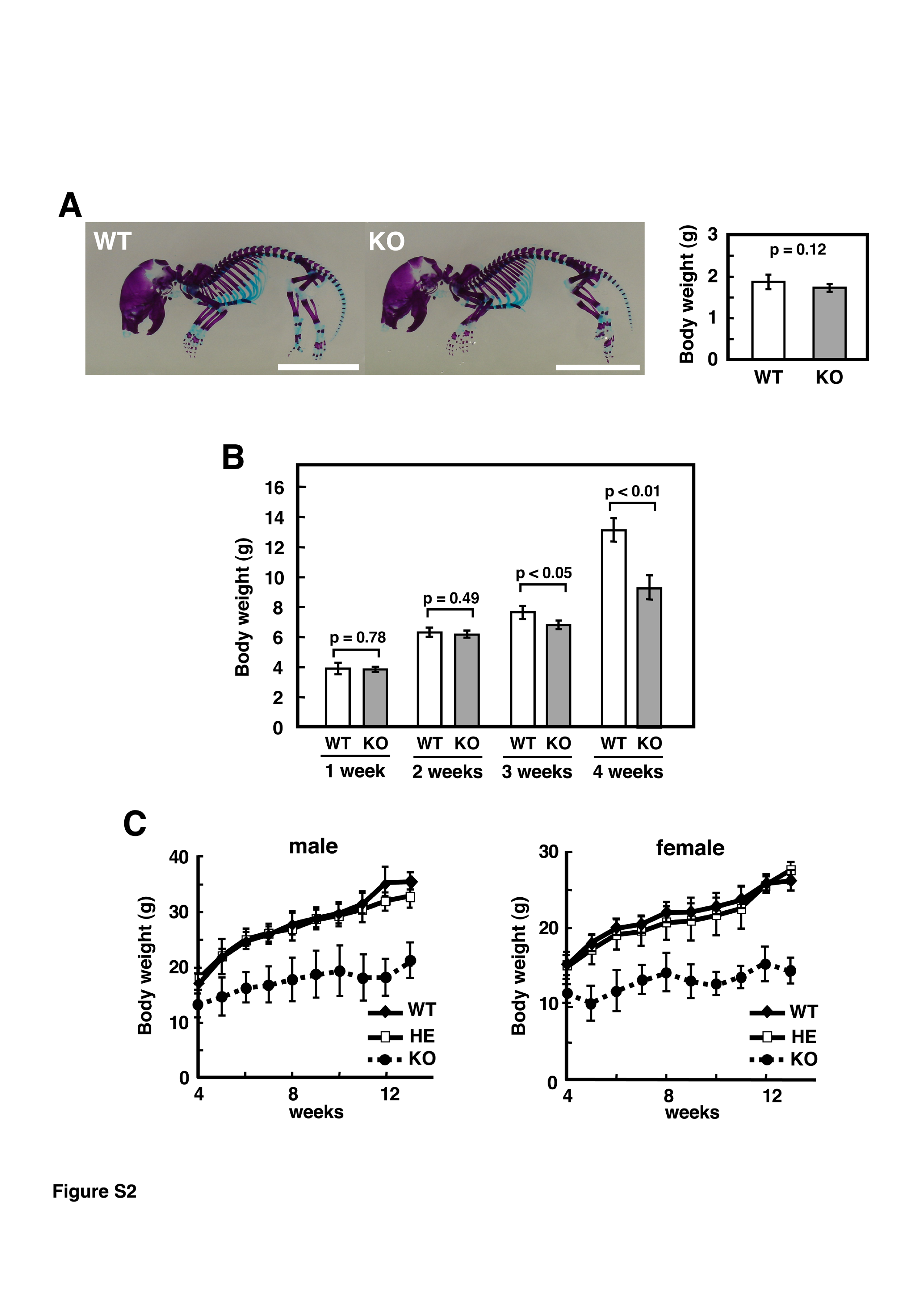

Supplement: Figure S2 — Growth retardation of Slc39a13-KO mice. A. Left: Little significant difference in skeletogenesis and body size at newborn between wild-type and Slc39a13-KO mice. Bar indicates 1 cm; Right: Body weight of newborn mice (n = 5 for each). Data represent mean±S.D. B. Significant delayed growth is observed after 3 weeks of age (n = 5; male mice). Data represent mean±S.D. C. Both male and female Slc39a13-KO mice show growth retardation (n = 10 for each). Data represent mean±S.E.M. WT: wild-type mice, HE: heterozygote mice, KO: Slc39a13-KO mice. (1.41 MB TIF) [file pone.0003642.s003.tif]

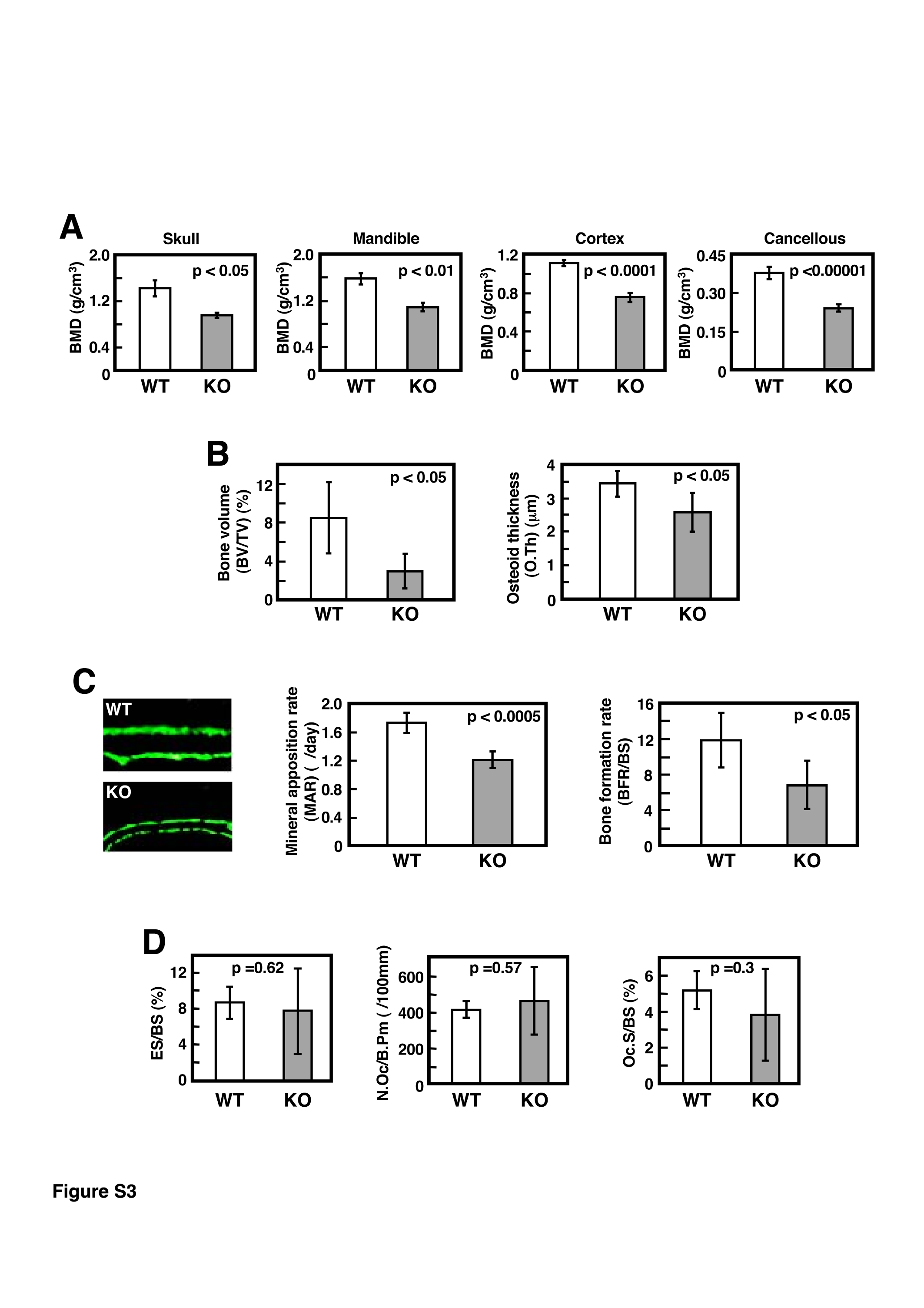

Supplement: Figure S3 — Skeletal histomorphometry of Slc39a13-KO mice. A-D: Bone histomorphometric analysis. Data represent mean±S.D. A. BMD (bone mineral density) in skull, mandible, cortex, and cancellous zone of femur are decreased in 5-week-old Slc39a13-KO mice compared with wild-type mice (n = 5 for each). B. Bone volume and osteoid thickness of 4-week-old Slc39a13-KO mice are lower than those of wild-type (n = 5 for each). C. Osteoblast function is significantly decreased in Slc39a13-KO mice. A double-labeling analysis of calcein (left), mineral apposition ratio (middle), and first and second calcein bone formation rate (right) of 4-week-old mice (n = 5 for each). D. Osteoclast activity of Slc39a13-KO mice is equivalent to wild-type littermates. Eroded surface (left), osteoclast number (middle), and osteoclast-covered bone surface (right) of 4-week-old Slc39a13-KO and wild-type mice are shown (n = 5 for each). (0.94 MB TIF) [file pone.0003642.s004.tif]

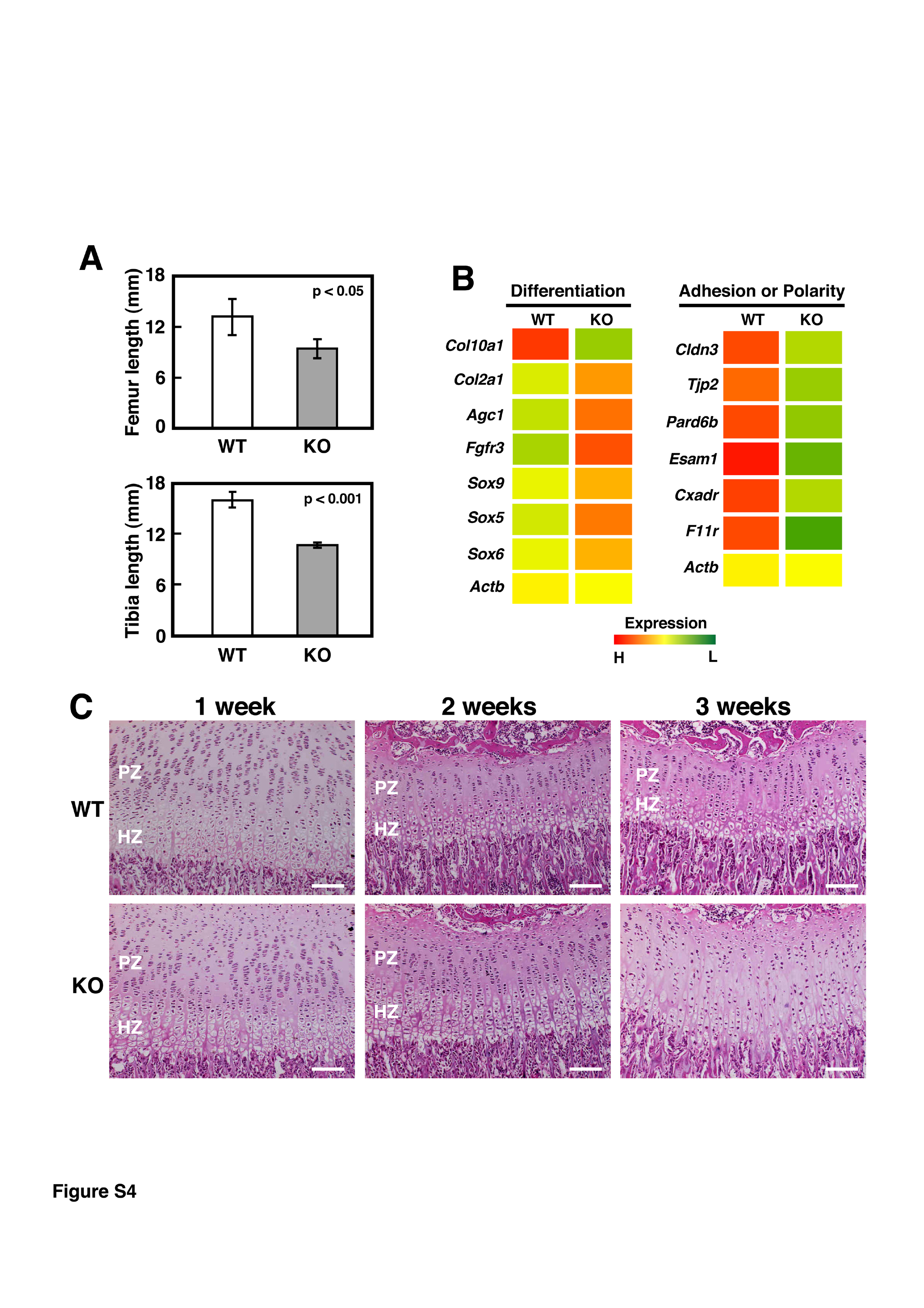

Supplement: Figure S4 — Abnormal cartilage development in Slc39a13-KO mice. A. Length of femur (upper) and tibia (lower) of 4-week-old Slc39a13-KO mice are shorter than those of wild-type (n = 5 for each). Data represent mean±S.D. B. Genes involved in chondrocyte differentiation (left) and in cell adhesion or polarity (right) are dysregulated in Slc39a13-KO primary chondrocytes. Gene expression profiling by DNA microarray analysis was carried out using total RNA from Slc39a13-KO or wild-type primary chondrocytes. Each gene was normalized to the median of the measurement for that gene. C. Abnormal morphology of growth plate in Slc39a13-KO mice. H&E staining images are shown. PZ: proliferative zone. HZ: hypertrophic zone. Bar indicates 100 µm. (4.46 MB TIF [file pone.0003642.s005.tif]

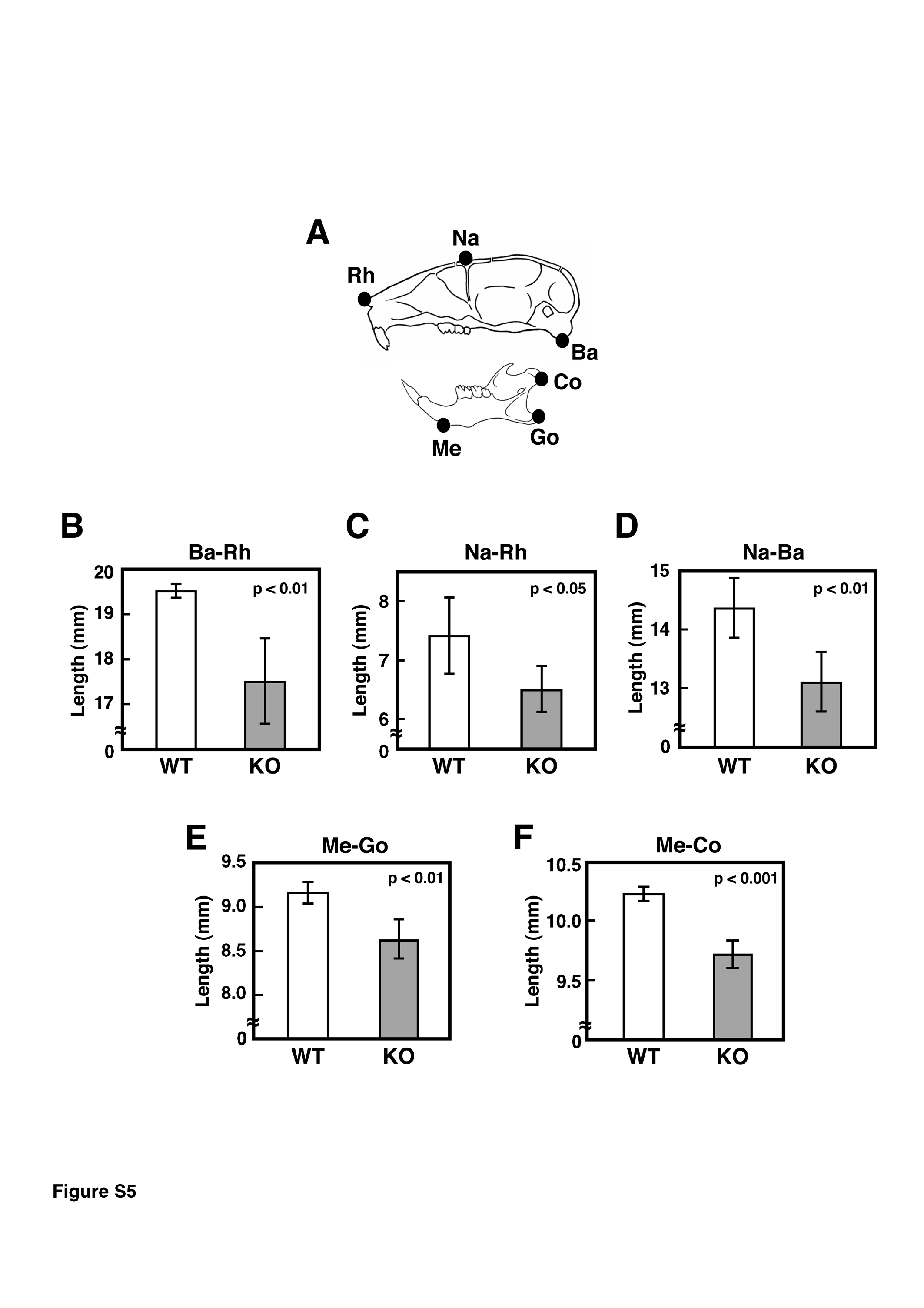

Supplement: Figure S5 — Impaired craniofacial skeletogenesis in Slc39a13-KO mice. A. The following points are traced on the cephalometric radiographs: Upper; Ba, Basion, defined as the most posterior-inferior cephalometric maxilla point; Rh, Rhinion, the most anterior point of the nasal bone; and Na, Nasion, the cephalometric point between the nasal bone and the frontal bone. Lower; Me, Menton, the lowest point of the chin; Go, Gonion, the most posterior inferior point at the angle of the mandible; and Co, Condylion, the most posterior superior point on the condyle of the mandible. B-F. Dwarfed craniofacial formation in Slc39a13-KO mice. Analysis of the 5-week-old maxilla and mandible bone reveals that Slc39a13-KO mice are characterized by shorter craniofacial depth (B. Ba-Rh), shorter nasomaxillary depth (C. Na-Rh), shorter cranial base depth (D. Na-Ba), shorter mandibular depth (E. Me-Go), and shorter craniofacial depth (F. Me-Co), compared with wild-type mice (n = 5 for each). Data represent mean±S.D. (0.75 MB TIF) [file pone.0003642.s006.tif]

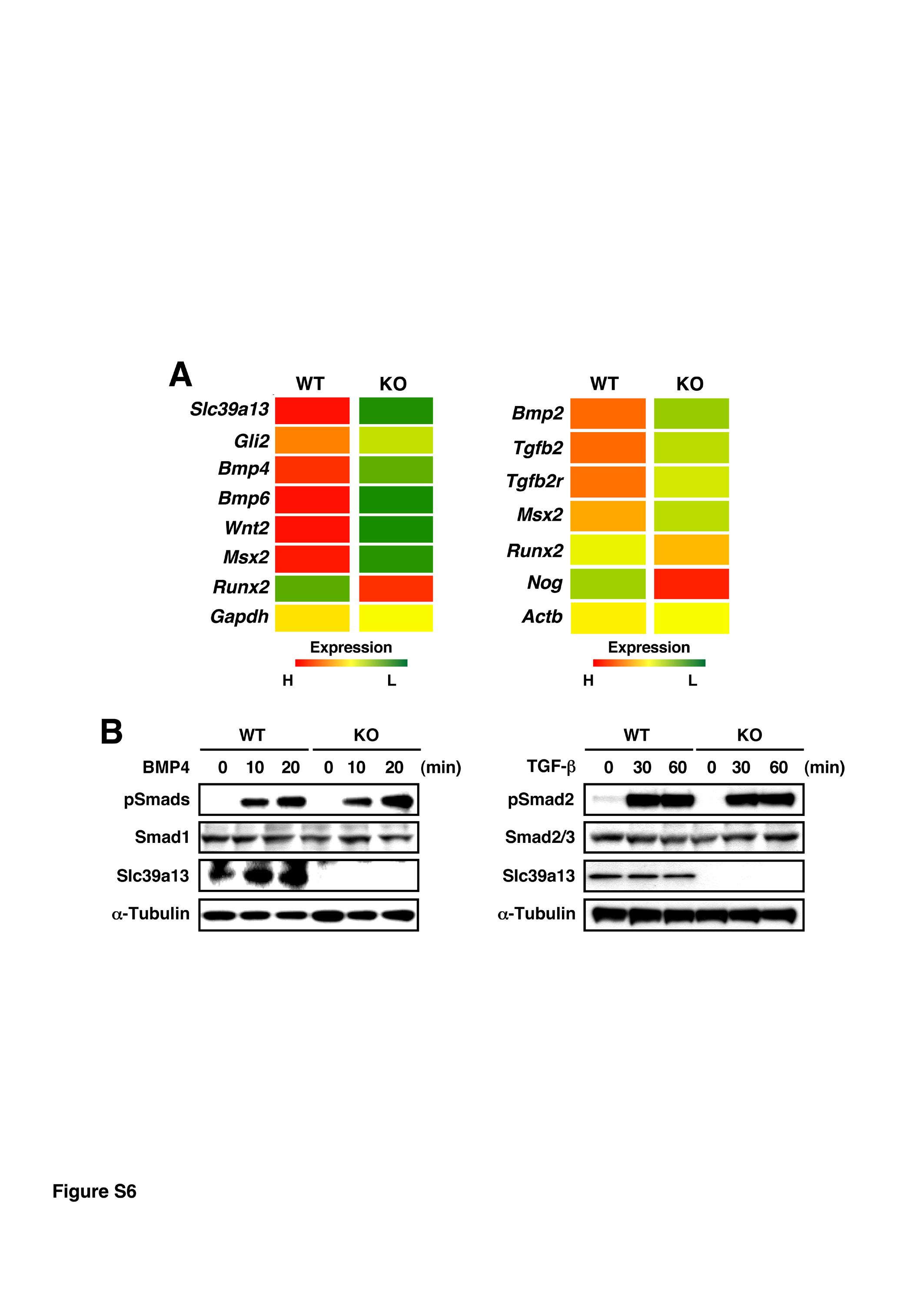

Supplement: Figure S6 — Perturbation of BMP/TGF-β signal transduction without affecting phosphorylation of Smad proteins in Slc39a13-KO cells. A. DNA microarray analysis using RNA of primary osteoblasts (left) and chondrocytes (right). Each gene was normalized to the median of the measurement for that gene. B. Smad proteins are normally phosphorylated in Slc39a13-KO cells. Primary osteoblasts or dermal fibroblasts were stimulated with either 50 ng/ml of BMP4 (left), or 10 ng/ml of TGF-β1 (right), respectively for indicated periods. Total cell lysates were separated by SDS-PAGE, followed by immunoblotting with either anti-phosphorylated Smad1/5/8 (pSmads), anti-Smad1, anti-phosphorylated Smad2 (pSmad2), anti-Smad2/3, or anti-Scl39a13 specific antibodies. Anti- α -tubulin antibody was used for control blotting. (0.84 MB TIF) [file pone.0003642.s007.tif]

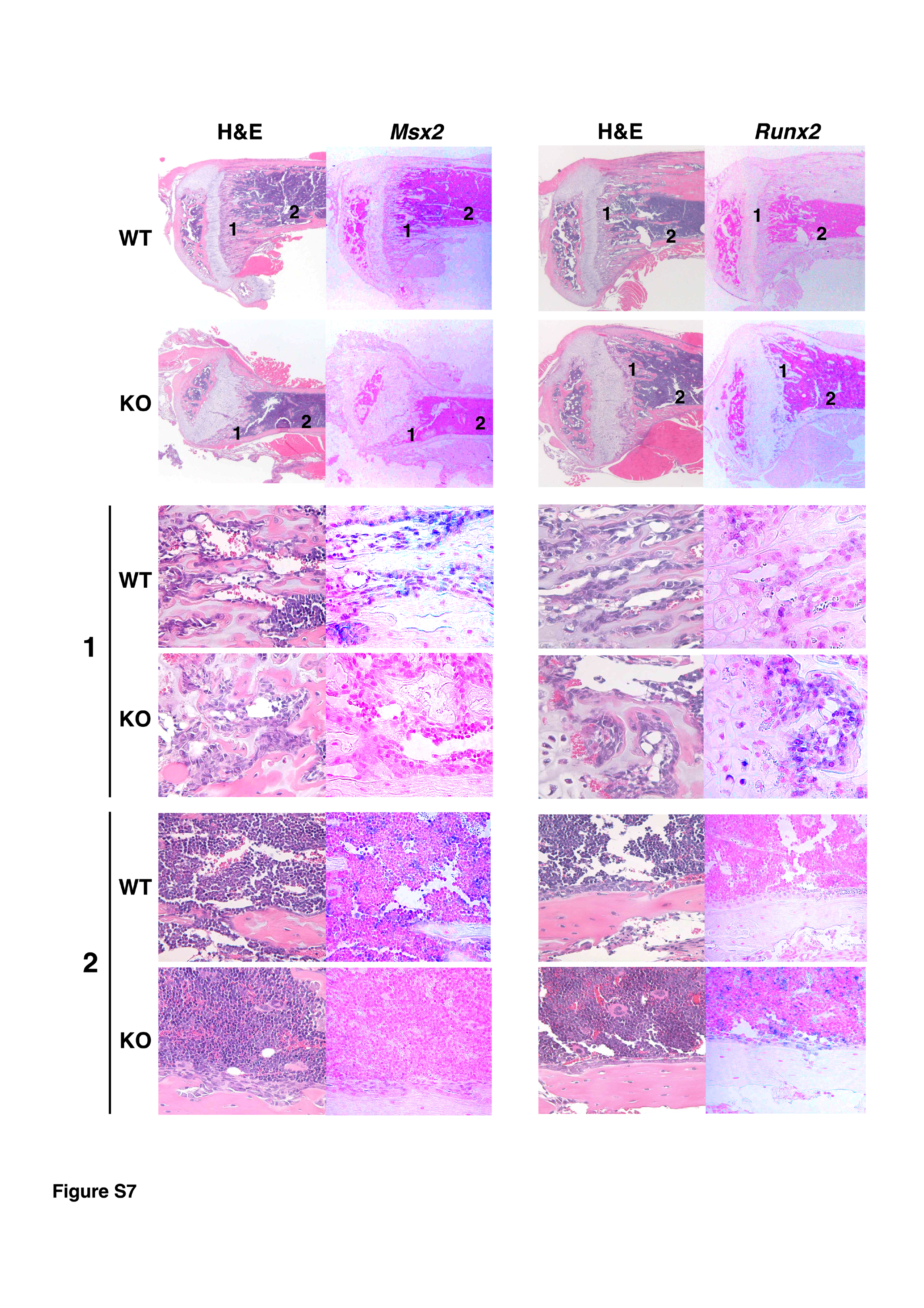

Supplement: Figure S7 — Dysregulated expression of tibial Msx2 and Rnux2 in Slc39a13-KO mouse. ISH analysis shows Runx2 is accumulated (right), while Msx2 is diminished (left) in 4-week-old Slc39a13-KO tibia. Regions indicated as 1 and 2 in upper are enlarged as 200 times at middle and lower panels. (8.41 MB TIF) [file pone.0003642.s008.tif]

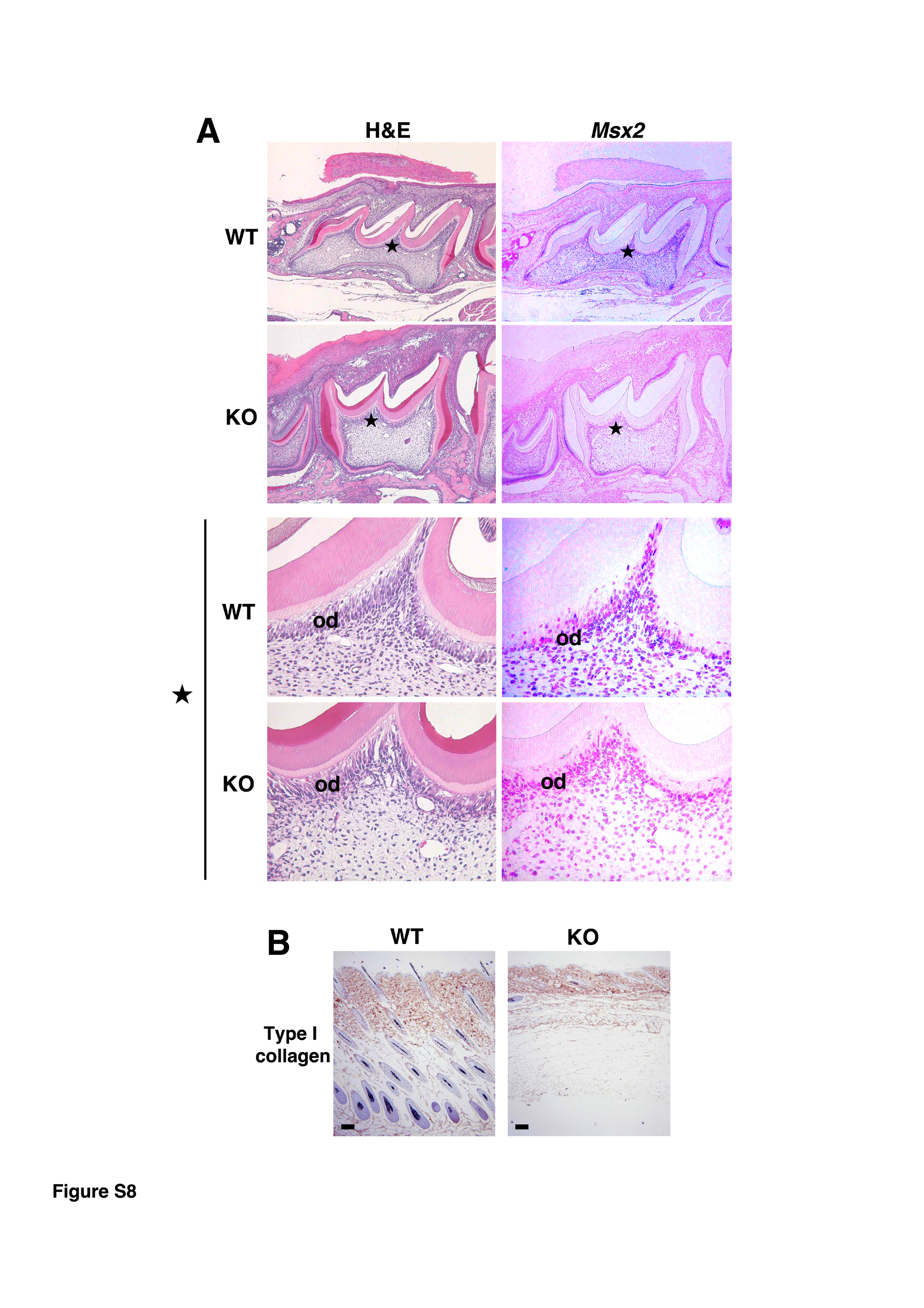

Supplement: Figure S8 — Dysregulated expression of molar Msx2 and dermal type 1 collagen expression in Slc39a13-KO mouse. A. ISH analysis shows Msx2 gene expression is diminished in odontoblasts (od) lining the dentin of crown (★) of 10-day-old Slc39a13-KO molar teeth. Regions indicated with ★ in upper are enlarged as 200 times at lower panels. Unorganized odontoblasts are observed in Slc39a13-KO molar. B. Type I collagen level is decreased in Slc39a13-KO skin. Skin section of 5-week-old wild-type and Slc39a13-KO mice were applied for IHC. Bar indicates 100 µm. (5.71 MB TIF) [file pone.0003642.s009.tif]

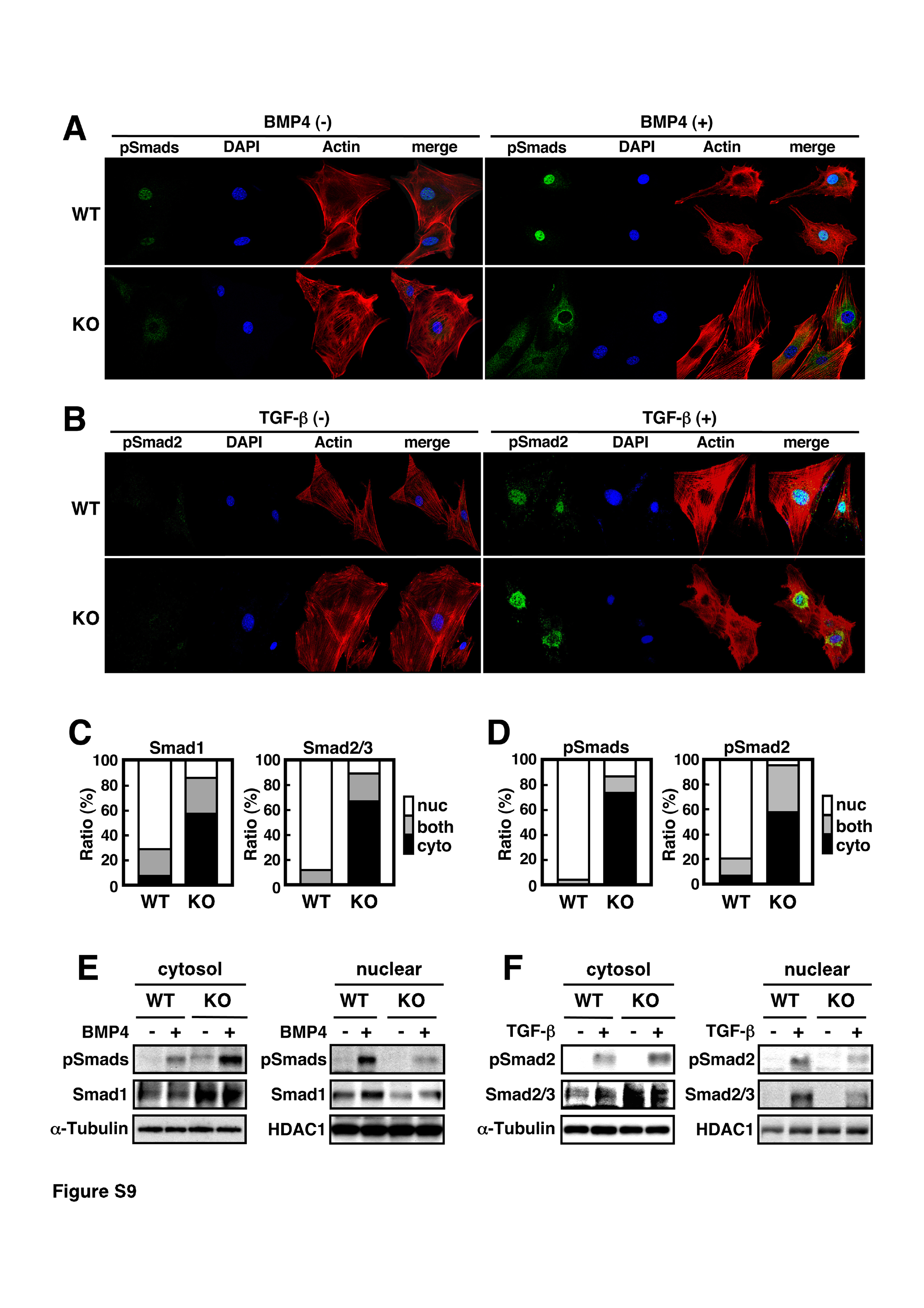

Supplement: Figure S9 — Involvement of Slc39a13 in localization of Smad proteins. A and B. Slc39a13 is involved in BMP/TGF-β -induced nuclear localization of phosphorylated Smad proteins. Primary osteoblasts (A) or dermal fibroblasts (B) were stimulated with either 50 ng/ml of BMP4 for 15 minutes (A, right panels) or 10 ng/ml of TGF-β1 for 30 minutes (B, right panels), respectively, followed by staining for phosphorylated Smad1/5/8 (pSmads), phosphorylated Smad2 (pSmad2), nuclei (DAPI), and actin (Actin). Confocal microscopic images are shown. C and D. Ratio of subcellular localization of intact (C) or phosphorylated (D) Smad proteins was obtained by counting cells (n = 50) in confocal microscopy images visualized by anti- Smad1, anti-Smad2/3, anti-phosphorylated Smad1/5/8 (pSmad), or anti- phosphorylated Smad2 (pSmad2) antibodies after BMP4 (left in C and D; for Smad1 and pSmads in osteoblasts) or TGF-β1 (right in C and D; for Smad2/3 and pSmad2 in dermal fibroblasts) stimulation. nuc: nuclear space, cyto: cytoplasmic space, both: both of nuclear and cytoplasmic spaces E and F. Cytoplasmic and nuclear fractions from primary osteoblasts (E) and dermal fibroblasts (F) were separated by SDS-PAGE, followed by immunoblotting with either anti-phosphorylated Smad1/5/8 (pSmads) and anti-Smad1 (E), or anti-phosphorylated Smad2 (pSmad2) and anti-Smad2/3 (F) antibodies. Anti- α-tubulin and HDAC1 antibodies were used for control blotting. (2.36 MB TIF) [file pone.0003642.s010.tif]

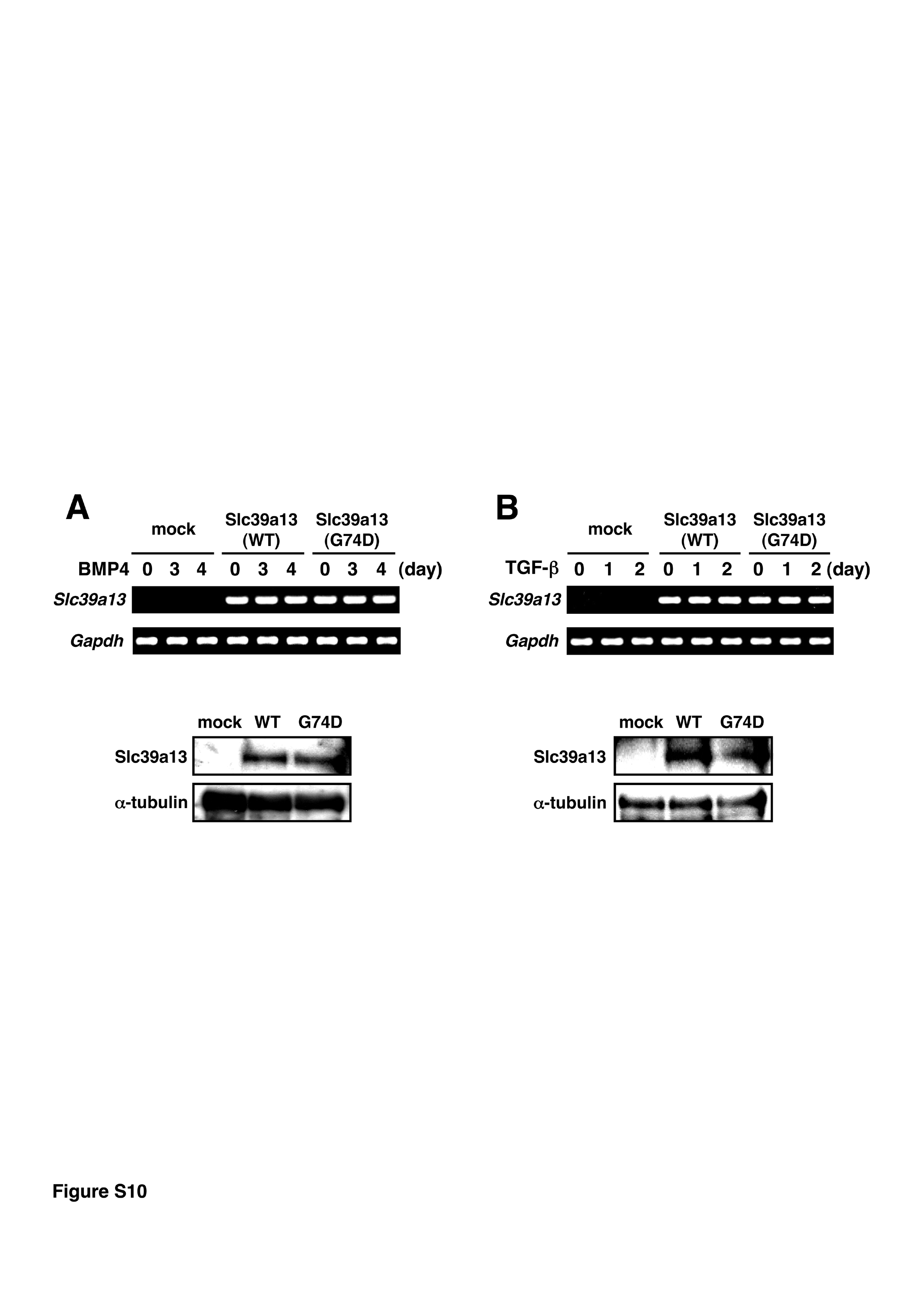

Supplement: Figure S10 — Ectopic expression of Slc39a13. Either empty vector (mock), Flag-tagged wild-type (WT), or G74D mutated (G74D) mouse Slc39a13 expression plasmids were transfected into Slc39a13-KO primary osteoblasts (A) and dermal fibroblasts (B). Their expression level was assessed by RT-PCR (upper), and by immunoblotting using anti-Flag and anti-α-tubulin antibodies at two days after transfection (lower). (0.72 MB TIF) [file pone.0003642.s011.tif]

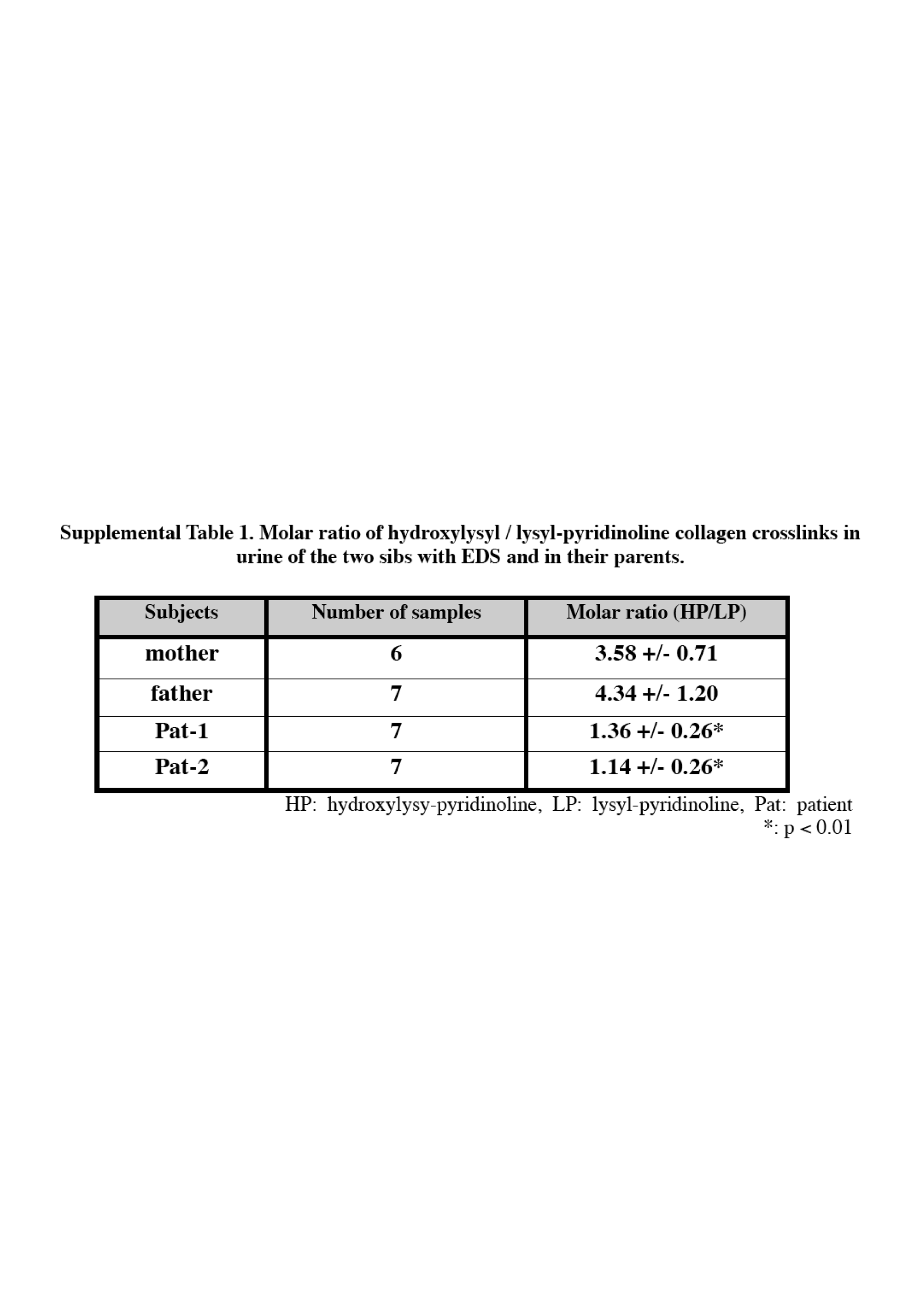

Supplement: Table S1 — Molar ratio of hydroxylysyl/lysyl-pyridinoline collagen crosslinks in urine of the two sibs with EDS and in their parents. (0.71 MB TIF) [file pone.0003642.s012.tif]

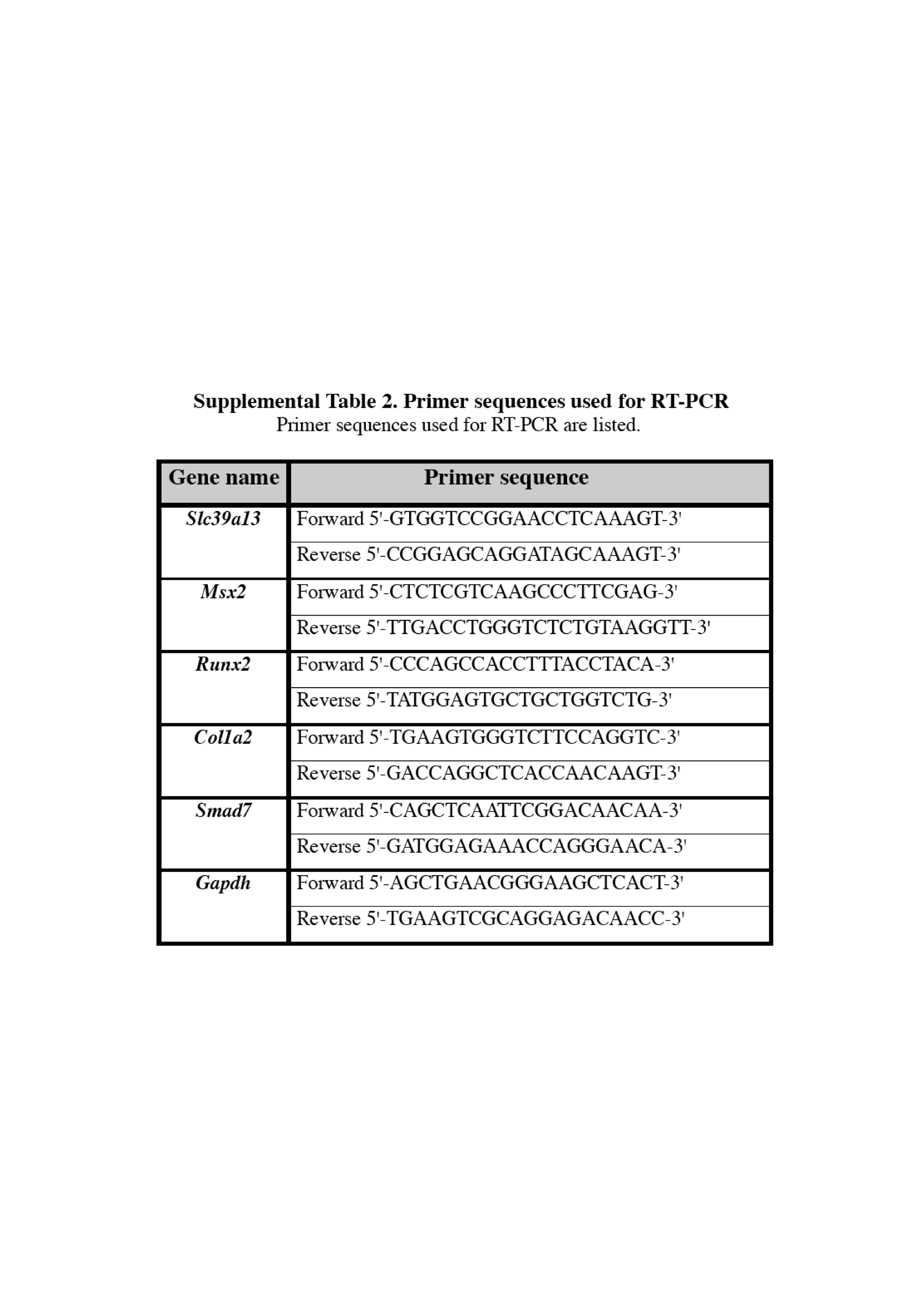

Supplement: Table S2 — Primer sequences used for RT-PCR are listed. (0.97 MB TIF) [file pone.0003642.s013.tif]

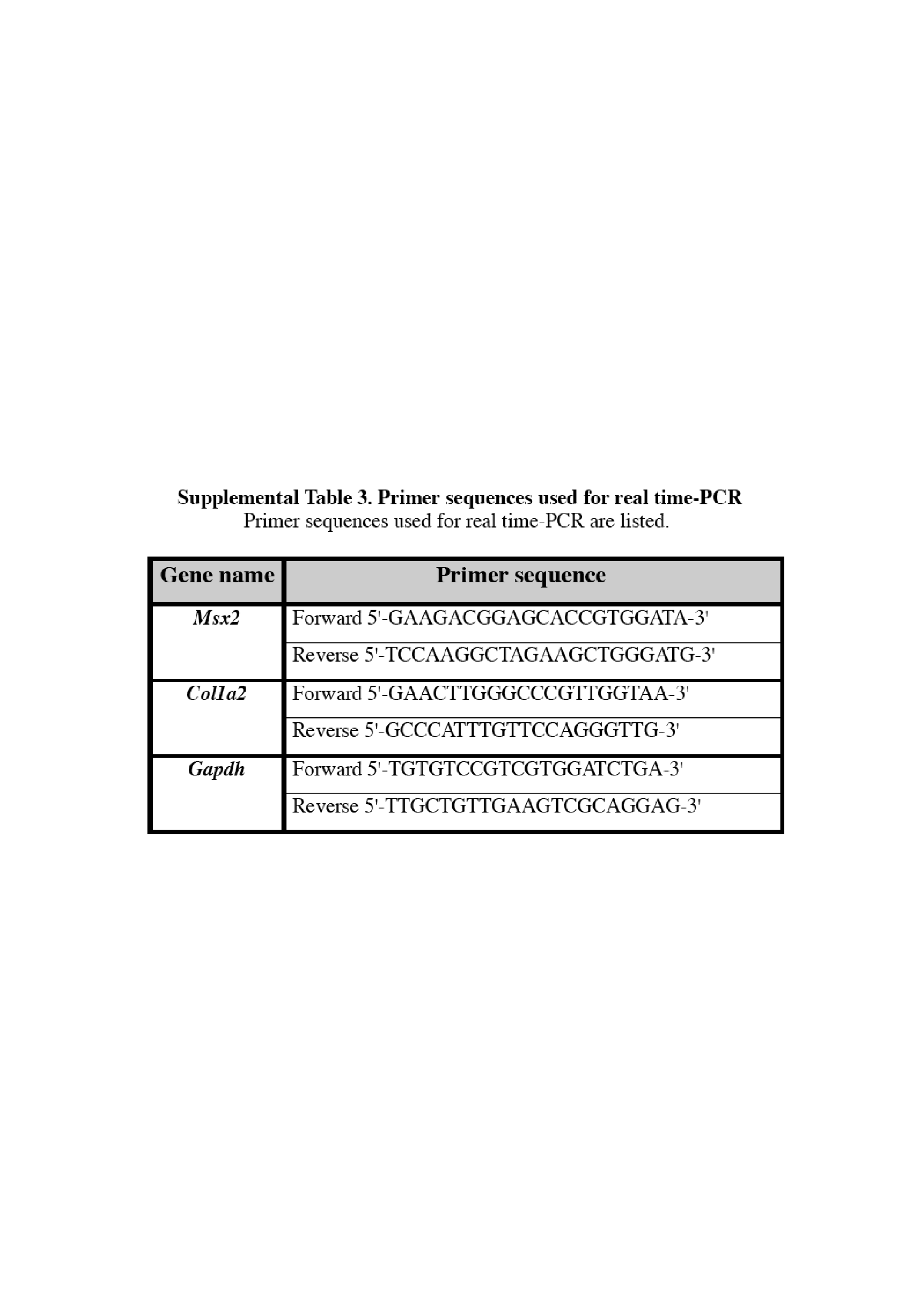

Supplement: Table S3 — Primer sequences used for real time-PCR are listed. (0.77 MB TIF) [file pone.0003642.s014.tif]
